# Multiscale Imaging Techniques for Real‐Time, Noninvasive Diagnosis of Li‐Ion Battery Failures

**DOI:** 10.1002/smsc.202300063

**Published:** 2023-10-08

**Authors:** Mingyu Lee, Jiwon Lee, Yewon Shin, Hongkyung Lee

**Affiliations:** ^1^ Department of Energy Science and Engineering Daegu Gyeongbuk Institute of Science and Technology (DGIST) 333 Technojungang-ro, Dalsung-gun Daegu 42988 Republic of Korea; ^2^ Energy Science and Engineering Research Center DGIST 333 Technojungang-ro, Dalsung-gun Daegu 42988 Republic of Korea

**Keywords:** battery diagnosis, current distribution, latent defects, lithium batteries, multiscale imaging techniques

## Abstract

With the increasing popularity of battery‐powered mobility, ensuring the safety and reliability of Li‐ion batteries (LIBs) has become critical for manufacturers. Despite advanced manufacturing processes for large‐scale Li‐ion cells, “latent defects” still unintentionally appear, due to imbalanced battery design, invisible faults, and extreme operating conditions. These defects cause performance degradation and can even lead to battery fires. Hence, early detection of latent defects, along with understanding the influence of cell parameters and operating conditions on battery failure scenarios, is crucial. For straightforward investigations and interpretations, noninvasive and in operando battery imaging techniques and methods have been proposed using X‐rays, neutrons, and ultrasound, as these can penetrate active and component materials and cell packaging. Moreover, magnetic‐field‐guided visualization of the current distribution pattern in cells under a current load has been proposed to identify invisible defects. This review thoroughly examines various imaging techniques for internal batteries, from the atomic and molecular levels in electrode materials and interfaces to macroscale battery systems. By assessing qualitative case studies and newly discovered phenomena, this review provides valuable insights into state‐of‐the‐art noninvasive battery imaging and its potential to improve the safety and reliability of LIB technology.

## Graphical abstract

Diagnosing Li secondary batteries using in operando, noninvasive imaging tools is crucial for securing their safety and reliability. Combining X‐rays, neutrons, ultrasound, and magnetic fields, multiscale battery imaging through its hierarchy from particle to cell level can provide a comprehensive picture of the structural and chemical information of the battery interior, aiding in understanding various failure scenarios and pinpointing the defect locations.

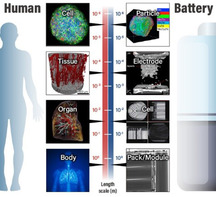

## 1 Introduction

As a global initiative aimed at reducing carbon emissions, the era of traditional combustion engines leads way to the rapid adoption of battery‐powered electric mobility. Since electric vehicles (EVs) rely on batteries as their power source, their performance depends heavily on the performance of these batteries. Consequently, batteries are the most crucial components of EVs. Over the past several decades, the battery industry has continually advanced, focusing on developing high‐output and high‐capacity lithium (Li)‐ion batteries (LIBs). These advancements aim to increase the range of EVs and reduce charging times and costs, thereby addressing the current limitations.^[^ ^1^, ^2^, ^3^ ^]^ Nonetheless, safety concerns remain unresolved.^[^ ^4^ ^]^ The recognition of the risks associated with EV safety has led to concerted efforts to enhance safety measures and address potential issues, as any safety incident can have significant negative impacts on the industry.^[^ ^5^, ^6^, ^7^, ^8^, ^9^, ^10^ ^]^

Most LIBs comprise a complexity of various materials and invlude the four main components of the cathode, anode, electrolyte, and separator. It is also composed of various materials from soft materials, such as packaging material and binder, to ceramics, carbon and metallic materials like a current collector, conductive additive, and external tabs.^[^ ^11^, ^12^ ^]^ Understanding the potential defects arising from the individual characteristics of each material and the degradation behavior within the battery is essential for validating safety and reliability.^[^ ^7^, ^13^ ^]^ Through extensive studies, the main origins of battery aging have been identified as the degradation of the crystal structure of active material^[^ ^14^, ^15^, ^16^ ^]^ and the chemical and electrochemical side reactions owing to the instability of the electrode/electrolyte interface.^[^ ^17^, ^18^, ^19^, ^20^ ^]^ These discoveries have provided insights into resolving problems in academia and industry and into validating performance reliability, e.g., by advancing manufacturing techniques.

However, accidental cell imbalances in performance‐oriented cell designs and upscaled manufacturing can increase the risk of battery failure and fire.^[^ ^21^, ^22^, ^23^, ^24^ ^]^ Unexpected faults or small errors are difficult to detect in the manufacturing process and can be considered “latent defects” that may emerge under extreme operating conditions.^[^ ^25^, ^26^, ^27^ ^]^ The term “latent” defects here refers to faults inside batteries that could not have been discovered by a reasonably thorough inspection before practical use. Although cell manufacturing processes have been intelligently automatized, it remains a challenge to determine the fault types and failure modes of cells and to seek the location of latent defects. For instance, several latent defects may include metallic impurities, such as Fe, Cu, and Al, within the cells that cannot be fully eliminated during manufacturing. Such metals could be eluted into the electrolyte from the current collectors and deposited at the electrode owing to abuse, such as over‐discharging and charging.^[^ ^28^ ^]^ Other latent defects include incomplete coverage of lean electrolytes, electrolyte contamination,^[^ ^29^ ^]^ and structural inhomogeneity of electrodes caused by local particle damage upon prolonged cycling.^[^ ^30^ ^]^

Such latent defects can disturb the homogeneous Li^+^ fluxes and electrical current distribution, rendering spots where accidental Li plating occurs and becoming a severe hazard during long‐term use, even under normal operating conditions.^[^ ^31^, ^32^, ^33^, ^34^, ^35^ ^]^ The factors causing unevenness in the current distribution, such as a local capacity imbalance among electrodes,^[^ ^36^, ^37^ ^]^ overcharging, fast charging, or an extremely low‐temperature environment, could lead to irregular plating on the electrode surface as the Li ions fail to be inserted into the graphite anode.^[^ ^38^, ^39^ ^]^ Furthermore, the transition metals (Ni, Co, Mn, etc.) eluted from the cathodes could be plated on the anode surface, causing localized Li plating.^[^ ^40^, ^41^, ^42^ ^]^ Excess Li plating, owing to its high reactivity, may promote side reactions in the electrolyte, deteriorated the solid‐electrolyte interphase (SEI), thereby accelerating performance degradation.^[^ ^31^, ^32^, ^43^ ^]^ Moreover, extensive Li plating can lead to dendritic growth, which can penetrate the separator and cause internal short circuits, leading to an increase in cell temperature and thermal runaway.^[^ ^44^ ^]^ Accordingly, the high risk of such latent defects has been recognized in commercial cell designs for large‐scale LIBs and next‐generation Li batteries (Li–S, Li‐metal, and solid‐state batteries).^[^ ^45^ ^]^ Nonetheless, there is a lack of understanding of the impact of latent defects on cell performance degradation or battery fire ignition scenarios.

Conventional post‐mortem and invasive analyses are limited in identifying the onset and specific location of latent defects and in the early diagnosis of battery degradation. Hence, it is of utmost importance to identify the effects of design factors and operating conditions (such as abuse, overcharging, abnormal temperature, and pressure) on battery degradation and explosion by detecting metal plating in advance through noninvasive analysis. Therefore, it is crucial to detect the latent defects arising in conditions after a current has been applied and to understand the mechanism(s) behind the consequent degradation. In traditional postmortem and invasive analyses, air exposure during battery disassembly can contaminate samples, leading to misinterpretation of results. Therefore, a noninvasive, in operando battery imaging analysis technology that enables the understanding of reaction dynamics in a nonequilibrium state under a current load without cell anatomy is required.

Whether the imaging technology is in situ or ex situ is determined by the transmissivity of the light source required for imaging and qualitative resolution. Ion beam, electron beam, UV‐visible, and IR imaging technologies are generally considered ex situ analyses requiring cell disassembly. Recently, a specially designed cell with a transparent or ultrathin window has been produced to conduct in situ analyses;^[^ ^38^, ^46^ ^]^ however, large deviation from the actual battery operating conditions poses limitations regarding result interpretation and application. In contrast, X‐ray, neutron, and ultrasonic imaging technologies exhibit high transmissivity to allow penetration through actual pouch cell packaging materials, as well as active materials and cell components; thus, studies have been actively investigating their use in in situ analyses.^[^ ^47^, ^48^ ^]^ Recently, a novel technology has been proposed that uses a magnetic field induced by an applied current was developed to visualize the current distribution pattern.^[^ ^49^ ^]^

**Figure 1:**
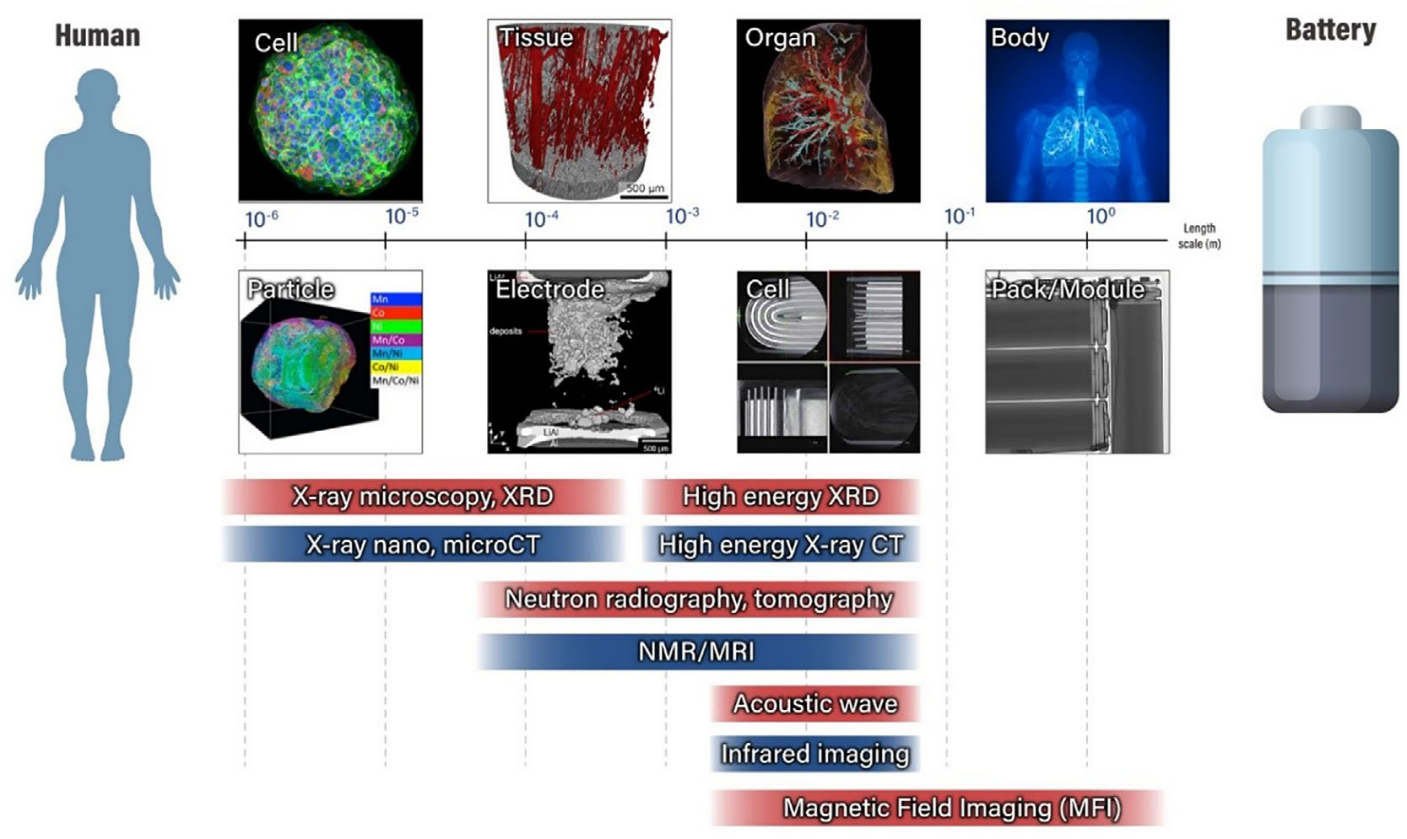
Schematic categories of in situ battery imaging techniques based on spatial resolution and detecting target scales (ranging from particle to battery pack) in Li‐ion battery systems. Analogous to medical imaging, which can visualize structural changes from stem cells to the human body, X‐ray, magnetic resonance imaging (MRI), acoustic wave imaging, and infrared cameras can be utilized to monitor the structural evolution within the cells upon an external current load. Unlike medical applications, neutron radiation can be used to chemically map the elements of interest, which is not feasible in medical applications. Apart from structural and chemical imaging, magnetic field imaging (MFI) has been proposed to globally visualize the electrical current distribution within the unit cells and at the cell arrays within the module or pack.

In this review, various imaging tools suitable for each scale (from active material particles to battery systems) are introduced, and their case studies and recently identified phenomena are discussed. In the field of medical radiology, spatial mapping of the human body down through its hierarchy, from entire organs to their individual functional units and constituent cells, can provide a comprehensive picture of the structural information and precise details of the entire intact human body, aiding in the diagnosis and treatment of diseases and injuries. Analogous to medical imaging (**Figure** **1**), this review aims to provide comprehensive guidance on appropriate imaging techniques across various length scales, ranging from the material level to the cell level, to detect defects and evaluate the internal states of batteries. Compared to other reviews, this review will pay more attention to imaging analysis at the cell and system levels, as well as imaging at the material level. Such an endeavor is important for finding locally and randomly distributed small flaws that could trigger catastrophic cell failures. Prior to embarking on an extensive examination at the material level, it is imperative to ascertain various failure modes and pinpoint the defect locations. This preliminary stage holds significant importance, as it serves as a fundamental step in the overall investigation. In this regard, this review further addresses the current flow imaging technologies that are expected to be helpful for sensing and detecting invisible defects that can trigger current abnormalities over the cells. By drawing an analogy to imaging of the human body and battery, this metaphor hopefully offers a fresh perspective on battery imaging technology, inspiring the exploration of innovative tools and methods.

## 2 Visualization of the Active Material Particle

### 2.1 X‐ray Microscopy/Diffraction Technologies

**Figure 2:**
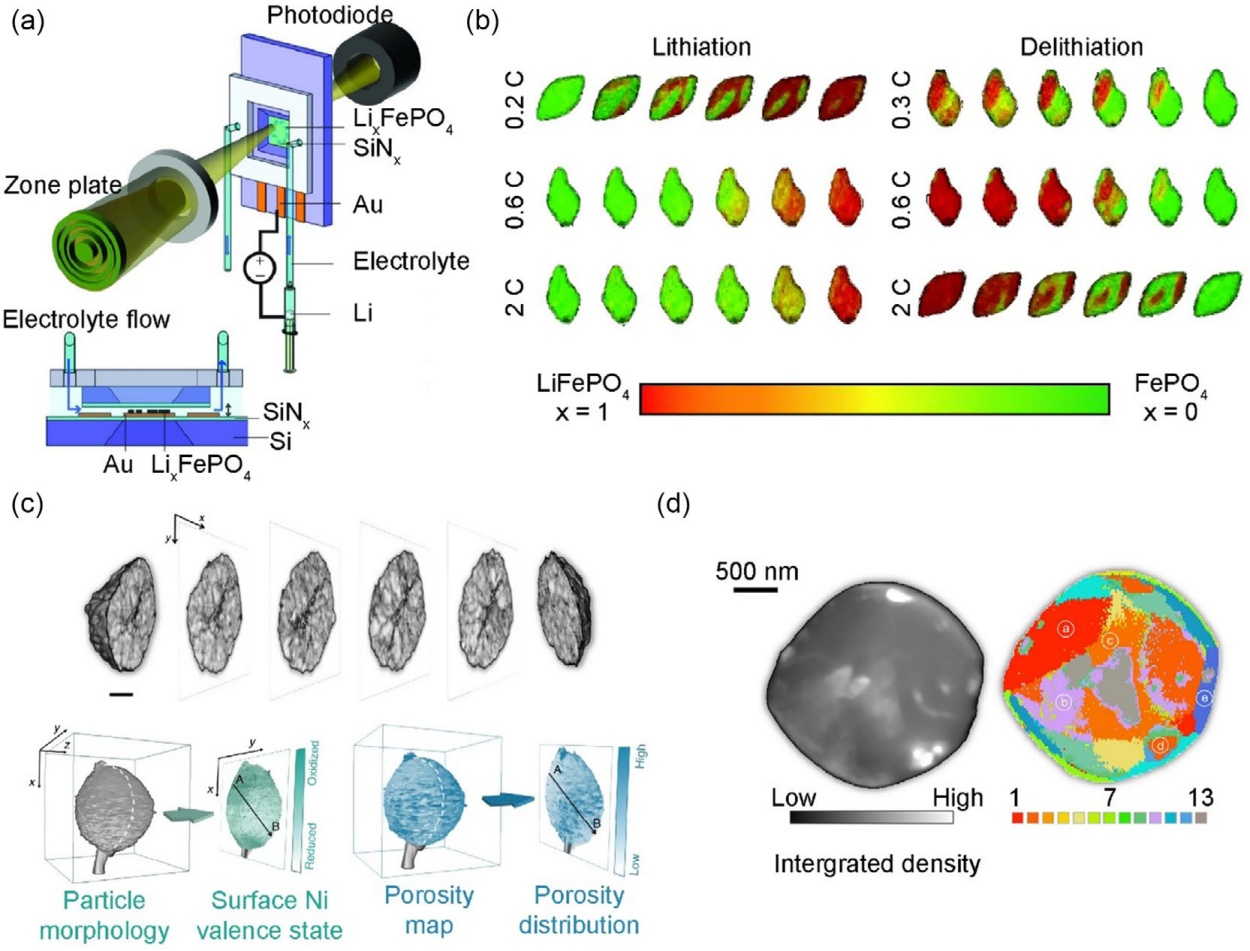
a) Scheme of the synchrotron‐based liquid scanning transmission X‐ray microscopy (SXTM) nanoimaging platform and b) selected operando STXM frames for Li insertion and extraction. a,b) Reproduced with permission.^[^ ^52^ ^]^ Copyright 2016, American Association for the Advancement of Science. c) 3D rendering of multi‐X‐ray probe images of the particle, surface Ni valence state distribution, and bulk porosity map of individual NMC811 particles. Reproduced under the terms of the CC‐BY Creative Commons Attribution 4.0 International license (https://creativecommons.org/licenses/by/4.0).^[^ ^54^ ^]^ Copyright 2020, The Authors, published by Springer Nature. d) Integrated diffraction intensity map and cluster map obtained by machine‐learning‐assisted diffractive imaging data analysis. Different clusters are color‐coded (1–13) through t‐stochastic neighbor embedding (tSNE), representing distinguishable lattice defects associated with different degree of distortion and local crystallinity. Reproduced with permission.^[^ ^56^ ^]^ Copyright 2022, Elsevier.

At the particle level, real‐time imaging of the chemical state of active materials provides valuable information on Li‐ion distribution and crystal evolution. X‐rays are capable of performing this analysis, possessing a wide range of wavelengths (0.1–120 keV) suitable for spectrometry and in situ analysis of the crystal structure and local structural changes in active materials. However, the X‐ray intensity decreases exponentially based on the sample density and thickness, necessitating an appropriate beam energy and sample thickness for effective image data.^[^ ^50^ ^]^ Numerous studies have utilized X‐ray diffraction (XRD) to visualize the distribution of Li ions based on their chemical state and location within a single active material particle, highlighting the value of this technique for battery research. Operando fluorescence‐yield X‐ray microscopy was proposed by Chueh et al. at Stanford University as a novel method for visualizing the state of charge (SOC) of LiFePO_4_ particles exposed to an electrolyte depending on the particle morphology (ellipsoidal or platelet like).^[^ ^51^ ^]^ They visualized the mosaic pattern arising from the variation in the Li‐ion distribution in an ellipsoidal particle where the Li ions were sequentially inserted, in contrast to a platelet particle with uniform insertion of Li ions. A novel route was suggested for Li insertion based on variations in the rate of the surface reaction in the particle morphology (which varied according to the synthetic conditions). In a follow‐up study, they further proposed the use of synchrotron liquid scanning transmission X‐ray microscopy to observe how the Li composition and insertion rate changed over time and space in platelet particles of LiFePO_4_.^[^ ^52^ ^]^ The visualization achieved a higher spatiotemporal resolution of the Li composition in single‐crystal Li_*x*_FePO_4_ particles during delithiation (charge) and lithiation (discharge) compared to the previous technique. Higher resolution was achieved by producing a customized cell with a microfluidic channel (**Figure** **2**a) and performing scanning transmission X‐ray micro‐scopy (STXM) analysis during the electrochemical operation (Figure 2b). Although the randomness of the Li composition in the Li_*x*_FePO_4_ particles increased upon delithiation, they reported for the first time the alleviation of irregular Li insertion with this imaging tool, demonstrating uniform Li insertion by alleviating the compositional changes in the particles at a high discharge rate.

In addition to visualizing the distribution of Li ions, obtaining a spatial mapping image of the local Ni valence state as the most redox‐active cation in the layer‐structured LiNi_1−*x*−*y*_Mn_*x*_Co_y_O_2_ (NMC) cathode is feasible for assessing the charge homogeneity at the particle level.^[^ ^53^ ^]^ Generally, Ni valence state changes in the spinel and/or rock‐salt phases can be a spectroscopic fingerprint in soft X‐ray absorption spectroscopy (XAS) data over the Ni *L*_3_‐edge, revealing the rearrangement of the layered lattice structure at high‐Ni NMC cathode surfaces. Liu et al. used multiple X‐ray probes in X‐ray microscopy imaging to investigate a single LiNi_0.8_Mn_0.1_Co_0.1_O_2_ (NMC811) particle in its charged state.^[^ ^54^ ^]^ Soft and hard X‐ray regimes were coupled with the energy tunability of the synchrotron source. As depicted in Figure 2c, by employing a scanning soft X‐ray nanoprobe, the surface Ni valence state is precisely mapped with a probing depth of ≈5 nm and a lateral spatial resolution of ≈30 nm to examine the chemical homogeneity at the particle surfaces. Full‐field transmission hard X‐ray microscopy (TXM) with a nominal spatial resolution (≈30 nm) reconstructs the interior of the particle, revealing the bulk characteristics of the particle. They found that regions with higher porosity were associated with more severe surface lattice reconstructions, elucidating the underlying interplay between charge heterogeneity, bulk fracture, and surface passivation at the single‐particle level.

X‐ray diffractive imaging is promising for identifying lattice defects and deformations within active material particles, which can offer new insights into optimal methodologies and rigorous exploration of suitable dopants in defect engineering.^[^ ^55^ ^]^ Liu et al. proposed an effective analysis method by integrating cutting‐edge X‐ray nanoprobe diffractive imaging and advanced machine learning models, enabling the precise characterization of lattice defects and deformations.^[^ ^56^ ^]^ This approach was employed to study the effects of annealing on a single‐crystalline LiCoO_2_ cathode material co‐doped with trace amounts of Ti, Mg, and Al. Successful direct visualization of mesoscale defect rearrangement was achieved (Figure 2d). The application of machine learning to the analysis of diffraction patterns and imaging has led to notable advancements in the study of lattice defects. Furthermore, future investigations are recommended to establish a deeper correlation between the diffraction patterns and specific types of lattice defects.

### 2.2 X‐ray Computational Tomography (X‐ray CT) Technologies

**Figure 3:**
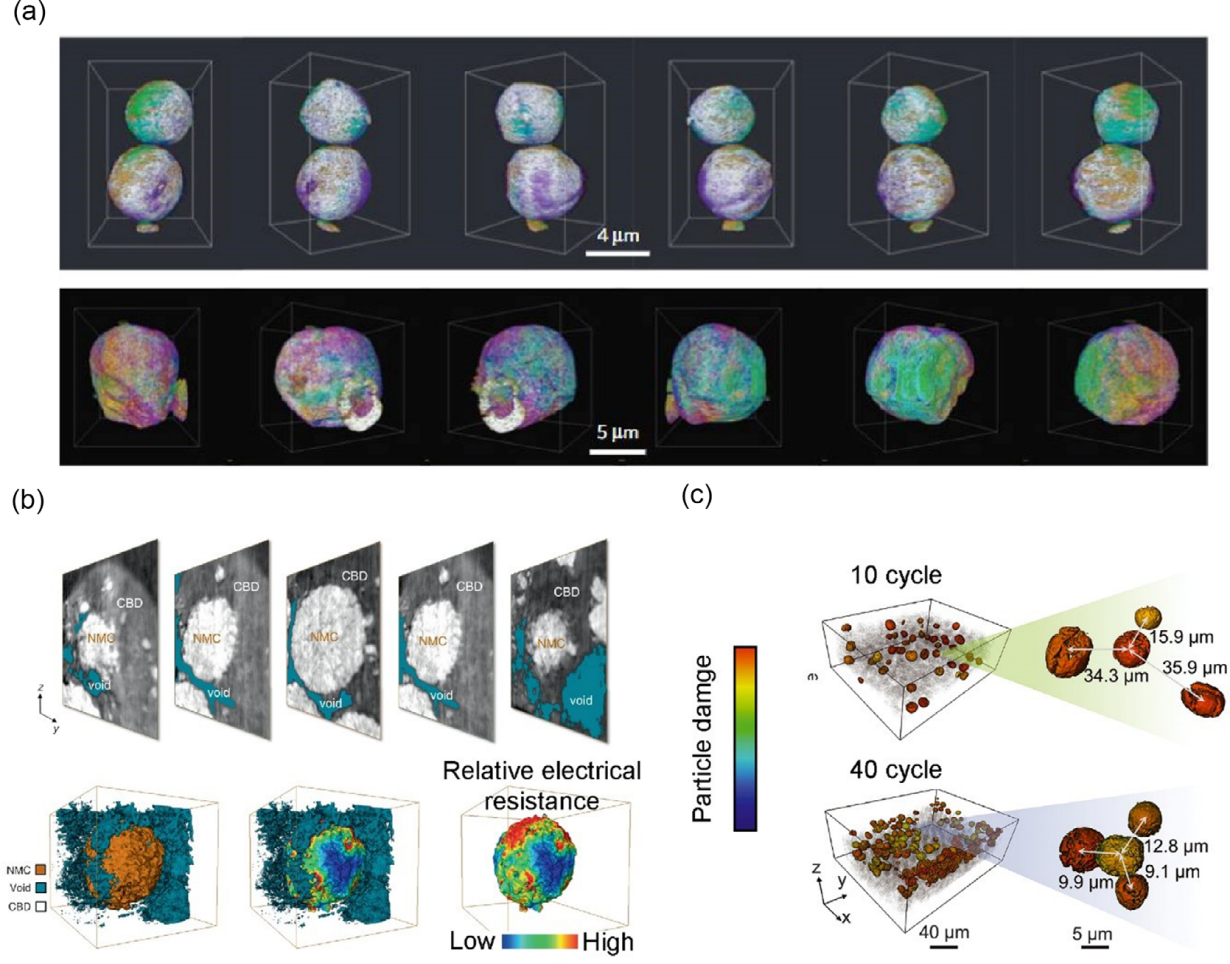
a) 3D rendering of the elemental association maps of NMC particle generated using X‐ray CT. Many nanodomains showed deviation in as‐made precursor and annealed NMC particles. Reproduced with permission.^[^ ^58^ ^]^ Copyright 2016, Springer Nature. b) 3D rendering of the segmentation results and distribution of relative local electrical resistance over the surface of NMC particle near the CBDs and voids. Reproduced under the terms of the CC‐BY Creative Commons Attribution 4.0 International license (https://creativecommons.org/licenses/by/4.0).^[^ ^59^ ^]^ Copyright 2020, The Authors, published by Springer Nature. c) Visualization of the spatial distributions of the damaged NMC particles and distance between the central particle and its three nearest‐neighboring damaged particles through X‐ray holotomography. Reproduced with permission.^[^ ^60^ ^]^ Copyright 2022, The Authors, published by American Association for the Advancement of Science.

X‐ray microscopy provides the evolution of the chemical state of active materials during battery operation yet is constrained with respect to the observation of physical changes in the battery component. Recently, computational tomography (CT), a three‐dimensional (3D) imaging technique widely used in medical diagnosis and industrial testing, has been used to analyze internal battery defects.^[^ ^57^ ^]^ By facilitating the observation of the structural evolution of electrodes, including expansion, contraction, crack formation, and phase changes, this technique was utilized to identify internal battery defects and investigate cell degradation mechanisms. For instance, Lin et al. successfully demonstrated 3D visualization of the transition metal distribution in a LiNi_0.4_Mn_0.4_Co_0.2_O_2_ (NMC442) material (≈10 μm) through full‐field hard X‐ray nanotomography.^[^ ^58^ ^]^ They examined the elemental 3D map of the as‐synthesized NMC442 surface and demonstrated that a Ni‐deficient and Mn‐enriched surface could be advantageous for a high‐voltage (≈4.7 V) operation (**Figure** **3**a). Direct measurement of the structural and chemical heterogeneity in the NMC cathode could offer valuable insights into the interplay among the local electrical resistance, particle morphology (size, shape, and cracks), arrangement of the ionic conductive network, and local potential gradient, which collectively affect the particle response to the operating conditions and subsequently influence cell chemistry.

Using quantitative X‐ray phase contrast nano‐tomography, Liu et al. visualized the detachment of NMC particles from the carbon‐binder domain (CBD).^[^ ^59^ ^]^ A numerical model was also developed to calculate the spatial heterogeneity of the electrical conductivity over the surface of the partially detached particles (Figure 3b). In particular, they leveraged a machine learning model that automatically identified and segmented more than 650 NMC particles. Their statistical analysis showed that fast‐cycled particles exhibited more severe detachment from the CBD, and smaller particles exhibited a higher degree of uncertainty in their CBD detachment. They further explored the possibility of utilizing the reconstructed local electron density as a proxy for the local SOC, highlighting the importance of balanced diffusion kinetics for both charge carriers (Li ions and electrons) to guide the design of future LIBs with optimal battery performance. Their statistical analysis through automatic segmentation and local resistance modeling could open the possibility of evaluating the intrinsic complexity of the morphology–performance relationship, such as particle size dependence, sphericity dependence, porosity dependence, and particle‐to‐particle interaction.

Recently, Liu et al. used hard X‐ray holotomography to visualize the structure of Ni‐rich NMC, such as an NMC811 composite cathode.^[^ ^60^ ^]^ By visualizing the overall picture of our statistical analysis of over 1000 particles and machine learning‐assisted tracking of the collective behavior over cycling, they found that the particles’ self‐attributes, together with the dynamic nature of the conductive network, jointly determined the damage behavior of the NMC particles in the composite electrodes (Figure 3c). In the early cycles, the individual particle characteristics predominantly determine their respective degrees of damage, featuring asynchronous behavior. However, in later cycles, the interplay among neighboring particles becomes more important, indicating that the local interparticle arrangement can critically affect the asynchronous‐to‐synchronous transition. In this study, tuning the shape and structure of the cathode particles, such as size, sphericity, and elongation, was suggested to suppress particle‐to‐particle variation in their structural characteristics. At the electrode scale, it was also suggested that an ordered electrode configuration with a tailored depth‐dependent, well‐defined packing of active particles would be robust for prolonged battery cycling.

Although in situ imaging studies with statistical analyses provide valuable insights into electrode engineering, they have several drawbacks. First, electrode damage during disassembly may skew the results of the statistical analysis. Second, electrode relaxation can cause charge redistribution, complicating the evaluation of potentially metastable electrode‐scale chemical heterogeneity. Third, cell‐to‐cell variations add complexity to the analysis and interpretation. These limitations can be addressed by using an operando experimental strategy. Lastly, single‐particle imaging techniques can only provide information on a small subset of particles within a battery and may not represent the overall battery behavior. Thus, single‐particle‐level imaging may not capture the degradation and failure mechanisms at the interfaces between different materials in the battery. Despite these limitations, single‐particle‐level imaging remains an important tool for understanding battery behavior and can provide valuable information on the chemical state and morphology of active materials. To gain a comprehensive understanding of battery behavior and failure mechanisms, these techniques are used in conjunction with larger‐scale imaging at the electrode level in the following section.

## 3 Visualization of Structural and Chemical Evolutions at the Electrode Level

### 3.1 X‐ray‐Based Imaging

**Figure 4:**
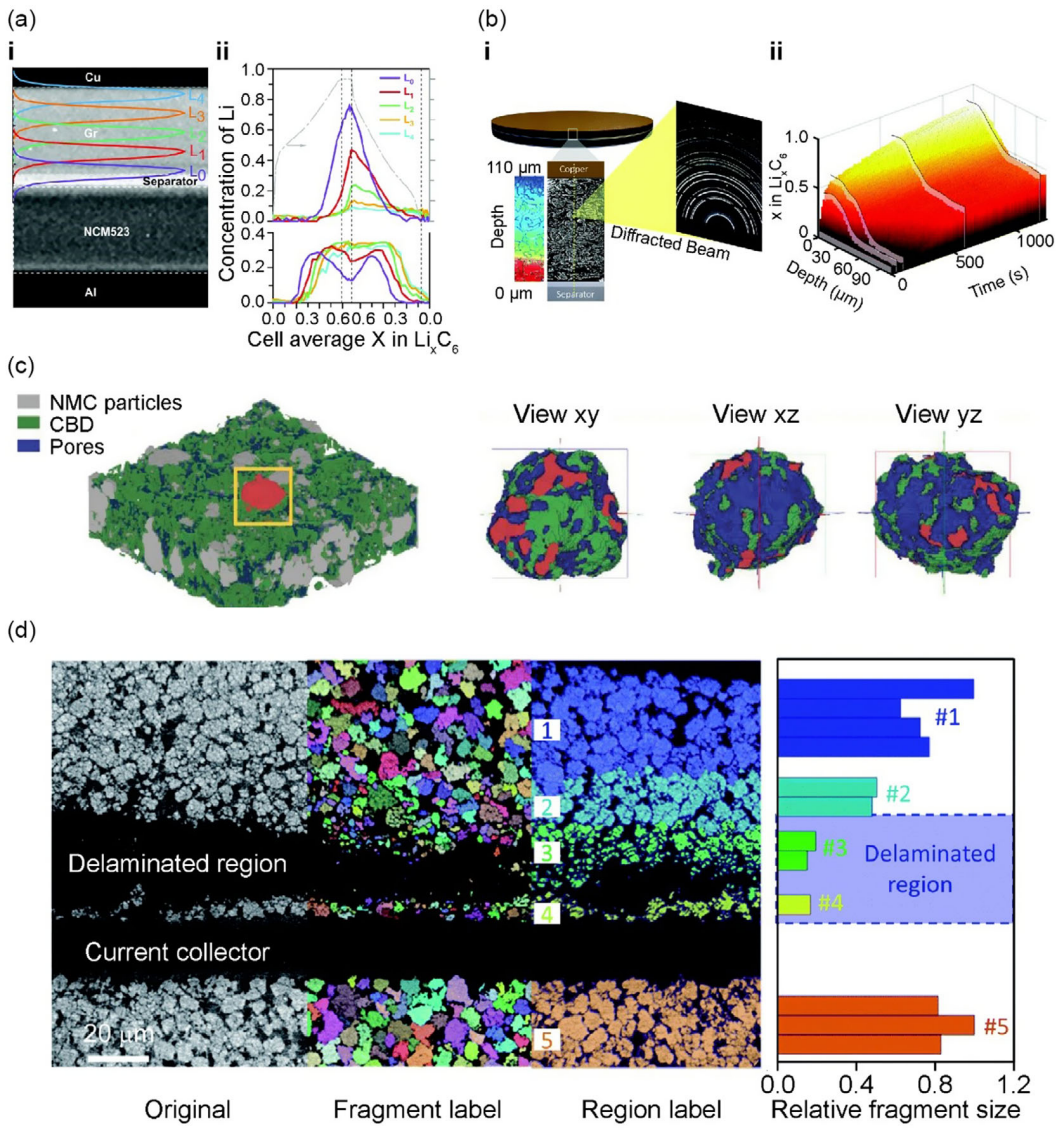
a) Energy‐dispersive XRD. i) Overlapping sections of layers (L_*n*_) and ii) average Li concentration of LiC_6_ and LiC_12_ phases at each layer during charge/discharge (1 C). a) Reproduced under the terms of the CC‐BY Creative Commons Attribution 3.0 Unported license (https://creativecommons.org/licenses/by/3.0).^[^ ^61^ ^]^ Copyright 2019, Royal Society of Chemistry. b) High‐speed pencil‐beam XRD. i) Negative (graphite) electrode with 35 point‐XRD measurements in 0.5 s and a lithiation gradient. ii) 3D and scatter plots of XRD point measurements of Li_*x*_C_6_ as a function of time and depth during 6 C charge. b) Reproduced under the terms of the CC‐BY Creative Commons Attribution 3.0 Unported license (https://creativecommons.org/licenses/by/3.0).^[^ ^62^ ^]^ Copyright 2020, Royal Society of Chemistry. c) 3D segmentation results and visualization of the individual NMC particle colored in red. Reproduced with permission.^[^ ^65^ ^]^ Copyright 2021, Wiley‐VCH. d) Quantification of the particle size distribution and delamination effect and the cracking of NMC particles are associated. Reproduced with permission.^[^ ^67^ ^]^ Copyright 2021, Royal Society of Chemistry.

As previously discussed, X‐ray‐based imaging has emerged as a powerful tool for assessing the charge state of individual particles in composite electrodes and investigating the homogeneity of composite electrodes. The Abraham group exploited energy‐dispersive XRD (ED‐XRD) to quantify the concentration of Li inserted into a graphite anode during the battery charge–discharge process (**Figure** **4**a).^[^ ^61^ ^]^ Through the diffraction pattern obtained at a fixed angle using a narrow focus beam of an ED‐XRD synchrotron photon, the Li_*x*_C_6_ (0 ≤ *x* ≤ 1) phase could be identified, and the peak intensity of each phase could be quantified, enabling the visualization of the uneven Li distribution in each graphite electrode (114 μm thickness). Although this technique allows the visualization of uneven Li distribution in each graphite electrode, its low spatial resolution and slow analysis speed (20 μm min^−1^) limit its further application. To address this issue, Smith et al. used high‐speed (100 Hz) pencil‐beam XRD to visualize Li insertion in a graphite anode during fast charging (10 min) in the direction of the electrode depth, improving spatial resolution and analysis speed (Figure 4b).^[^ ^62^ ^]^ Through high‐speed depth‐profiling synchrotron XRD to convert the image at 3 μm and 0.5 s intervals in a graphite electrode with a thickness of 101 μm, they improved the spatial resolution and analysis speed and found that the charging/discharging reaction preferentially occurs on the top surface of the graphite anode, and Li plating/stripping mainly occurs within 15 μm of the separator. Matsubara et al. presented a method for the nondestructive detection of Li electrodeposition in automotive prismatic cells using synchrotron XRD.^[^ ^63^ ^]^ By employing high‐energy X‐rays with a wavelength of 61 keV, the Li (110) peak area could be selectively determined to evaluate the relative quantity of Li. Furthermore, they examined the SOC distributions of the cathode by examining the (101) peak of the NMC111 diffraction patterns and revealed an uneven SOC distribution in the cathode, which was caused by local Li loss on the anode side driven by accidental Li plating, suggesting a possible origin of the cross‐deterioration between the cathode and anode.

In addition, high‐resolution XRD and computed tomography (XRD‐CT) can be applied to quantify the crystal heterogeneity within and between particles across the entire electrode structure. The Finegan group of NREL fabricated a special cell based on a Li_*x*_Mn_2_O_4_ cathode and Li metal. They quantified the stoichiometric differences among the particles of the Li_*x*_Mn_2_O_4_ material before and after cycling, revealing a stoichiometric gradient within the particles and phase heterogeneity.^[^ ^64^ ^]^ Most particles were shown to acquire an area of local conversion to LiMnO_2_ in the first cycle, and after 150 cycles, LiMnO_2_ and Li_2_MnO_3_ (as structures formed via Mn dissolution) could be spatially quantified. Despite significant advancements in X‐ray CT tools, it remains a challenge to identify the CBD in composite electrodes of LIBs, given the low attenuation coefficient of X‐rays. Demortière et al. proposed X‐ray nanoholotomography to quantify the microstructural properties of a LiNi_0.5_Mn_0.3_Co_0.2_O_2_ (NMC532) electrode.^[^ ^65^ ^]^ They were able to produce high‐resolution 3D images of the electrode microstructure and quantify important properties, such as porosity, tortuosity, and particle size distribution (Figure 4c). They found that the microstructural properties of the electrode significantly affected the electrochemical performance. The Ho group produced a special cell for performing high‐resolution in situ X‐ray microtomography to quantify random lithiation according to the electrode depth and Li plating on the electrode surface.^[^ ^66^ ^]^ Although the use of this technique had been limited in previous studies for Li plating on graphite owing to image noise and the low attenuation coefficients of Li and carbon, the limitation was overcome by using of a high‐resolution image that differentiated the graphite, pores, and Li‐metal phase. They found that fast charging caused Li plating on the graphite anode surface, which could lead to battery failure and safety concerns. Moreover, the SOC mapping based on the Δ*V*/*V* of the lithiated graphite suggested that the inhibition of Li insertion in the graphite particle was found at the position of the anode with Li plating.

Liu et al. presented a macro‐to‐nanoscopic overview of the hierarchical structure within a cylindrical cell with self‐discharging failures using a suite of X‐ray microscopy techniques, including microtomography, synchrotron phase‐contrast holotomography, and synchrotron nano‐resolution pectro‐tomography.^[^ ^67^ ^]^ Through nondestructive 3D X‐ray microtomography, they identified electrode delamination spots (Figure 4d). High‐resolution synchrotron imaging revealed that the primary cause was not the lack of cohesion between the NMC electrode and the Al current collector, but rather the cracking of active particles, leading to mechanical weakness and subsequent delamination.

### 3.2 Neutron‐Based Imaging

**Figure 5:**
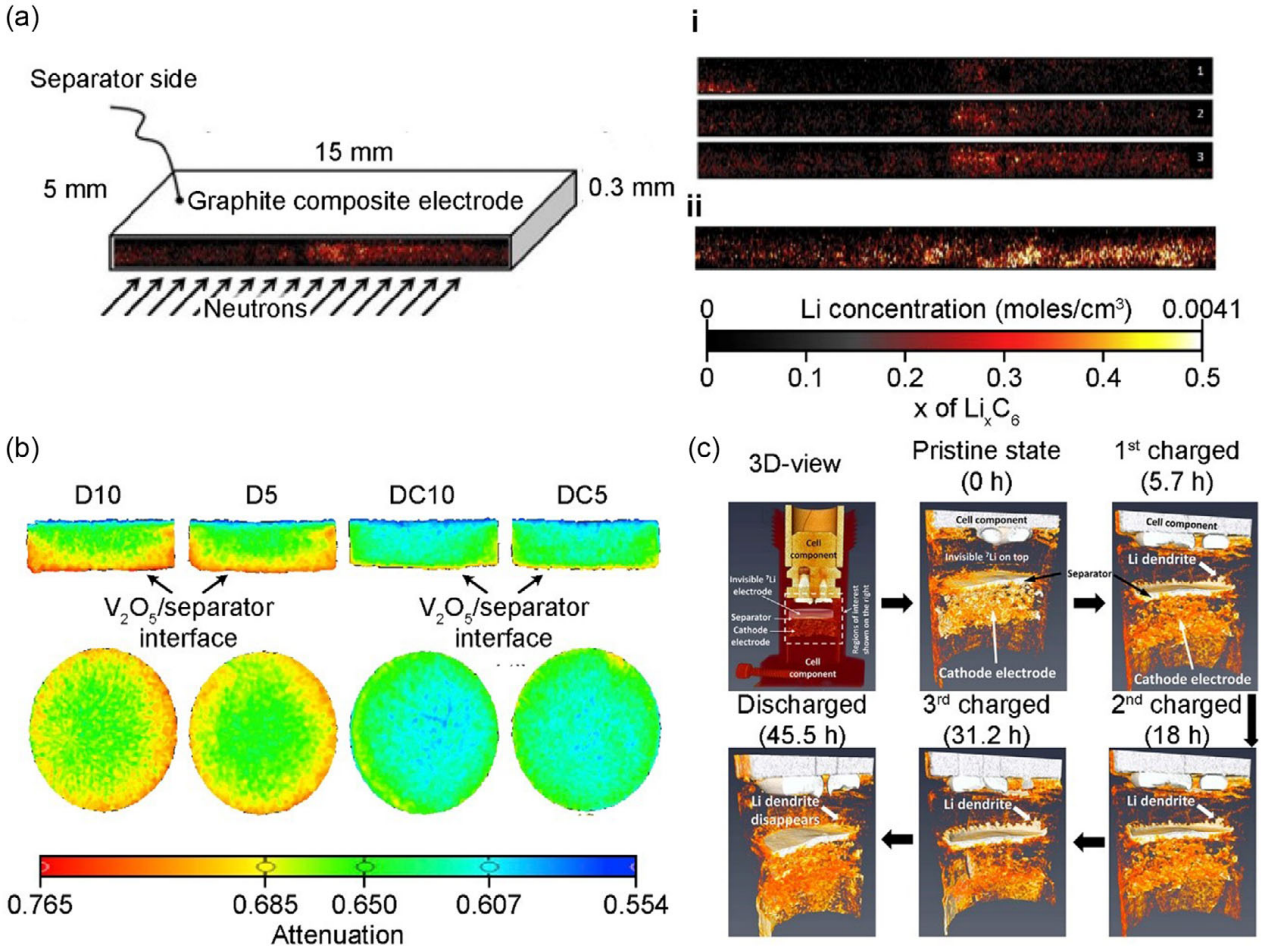
a) Neutron imaging of Li distributions in graphite anode: i) during the first discharge and ii) at fully charged state after the first cycle. a) Reproduced with permission.^[^ ^68^ ^]^ Copyright 2012, Elsevier. b) Neutron imaging data of sample discharged (D10 and D5) and recharged V_2_O_5_ electrode (DC10 and DC5). In this pseudo‐color plot, higher attenuation represents higher Li concentration. Reproduced with permission.^[^ ^70^ ^]^ Copyright 2017, Elsevier. c) 3D evolution of the Li distribution in the cell at different stages of charging and discharging. The white arrows in the series of images point at the dendritic Li around the disk edge of the ^7^Li electrode, growing during charge and vanishing at the end of discharge. Reproduced with permission.^[^ ^71^ ^]^ Copyright 2019, American Chemical Society.

Although X‐rays are useful for visualizing the uneven changes in the chemical state and structure of a single particle within the composite electrodes, they do not allow for the direct detection of hydrogen and Li. To overcome this problem, another study considered the high visibility of neutrons for Li and observed Li‐ion dispersion, electrolyte consumption, and gas formation through neutron‐based imaging. The Owejan group quantified Li inserted into a graphite electrode in a half‐cell using neutron radiography imaging.^[^ ^68^ ^]^ The capacity‐loss distribution was quantified by comparing the Li content before and after cycling. Significantly, the lost Li was mostly trapped in the bulk electrode material near the current collector, rather than contributing as irreversible Li by forming an SEI (**Figure** **5**a), which indicates that the lost Li was associated with areas of high local transport resistance. A similar technique to visualize the Li intercalation/deintercalation process according to variations in the current density during the battery charge/discharge process.^[^ ^69^ ^]^ It was confirmed that discharge capacity fading of the battery occurred owing to lithiation and delithiation within the vicinity of the separator at high current densities. In addition, the electrode tortuosity at a low current density had little effect on the charge–discharge performance.

Neutron‐based tomography was performed with a standard coin cell for 3D imaging of the spatial distribution of Li ions on the V_2_O_5_ electrode layer during the charge–discharge process (Figure 5b). A correlation between the quantitative distribution of Li ions and discharge capacity was shown, and an uneven distribution of Li ions being inserted in the V_2_O_5_ electrode at a high charge current was observed.^[^ ^70^ ^]^ They found an inconsistency in the Li‐ion distribution, which implies that Li‐ion diffusion within the electrode is restricted during the lithiation (discharge) process upon repeated cycling at high C‐rates. Understanding the effects of Li plating on graphite anodes on the performance and safety of LIBs and the deposition and growth of Li dendrites in next‐generation Li‐metal batteries are essential for improving battery performance. Thus, Song et al. visualized dendrite growth and “dead” Li formation in the charge–discharge process using neutron tomography (Figure 5c). A dynamic distribution of Li ions flowing from the anode to the cathode during charging, induced by an internal short circuit according to the growth of dendrites, was observed.^[^ ^71^ ^]^ Furthermore, a mechanism that competes between short‐circuit‐induced self‐discharging and charging has been proposed to explain the drop and rise in voltage during extended charging periods after an internal short circuit occurs.

Various battery imaging techniques, including X‐ray and neutron imaging, have limitations with respect to their ability to detect specific elements. Therefore, the coupling of multiple imaging methods can provide a better understanding of battery degradation. For instance, neutrons are sensitive to Li and H, whereas X‐rays are more sensitive to elements with higher atomic numbers such as Cu. By combining these two techniques, it is possible to perform a more in‐depth analysis of electrodes and electrolytes in batteries. The integration of neutron and X‐ray imaging modalities, termed neutron‐ and X‐ray‐based tomography (NeXT), was proposed by Weker et al.^[^ ^72^ ^]^ as a robust and nondestructive imaging platform. In particular, they focused on the ex situ 3D visualization of graphite anode degradation under extreme fast charging (XFC) conditions. By capitalizing on the distinct sensitivities of neutrons to low‐atomic‐number (Z) materials such as Li and X‐rays to high‐Z materials such as Cu, NeXT enables comprehensive results of the morphological and compositional changes in graphite anodes before and after XFC cycling. The simultaneous acquisition of neutron and X‐ray data from the same sample locations facilitates precise material identification and segmentation, thereby showcasing the inherent advantages of this integrated imaging approach.

### 3.3 Magnetic Resonance Imaging (MRI)

Visualization of the SOC at the electrode level and dendrite growth on the electrode surface can be achieved using neutron radiography and tomography techniques, which measure the neutron beam absorbed and attenuated by Li. However, these techniques have limitations due to the low atomic density of Li in the electrolyte, which hinders the measurement of changes in Li‐ion homogeneity and concentration. In contrast, nuclear magnetic resonance (NMR) analysis enables the measurement of the Li ion concentration in the electrolyte and the differentiation between the signal of bulk Li metal and that of Li in microstructural plating such as dendrites. Additionally, NMR facilitates 3D image conversion through magnetic resonance imaging (MRI), allowing the facile visualization of battery components.

**Figure 6:**
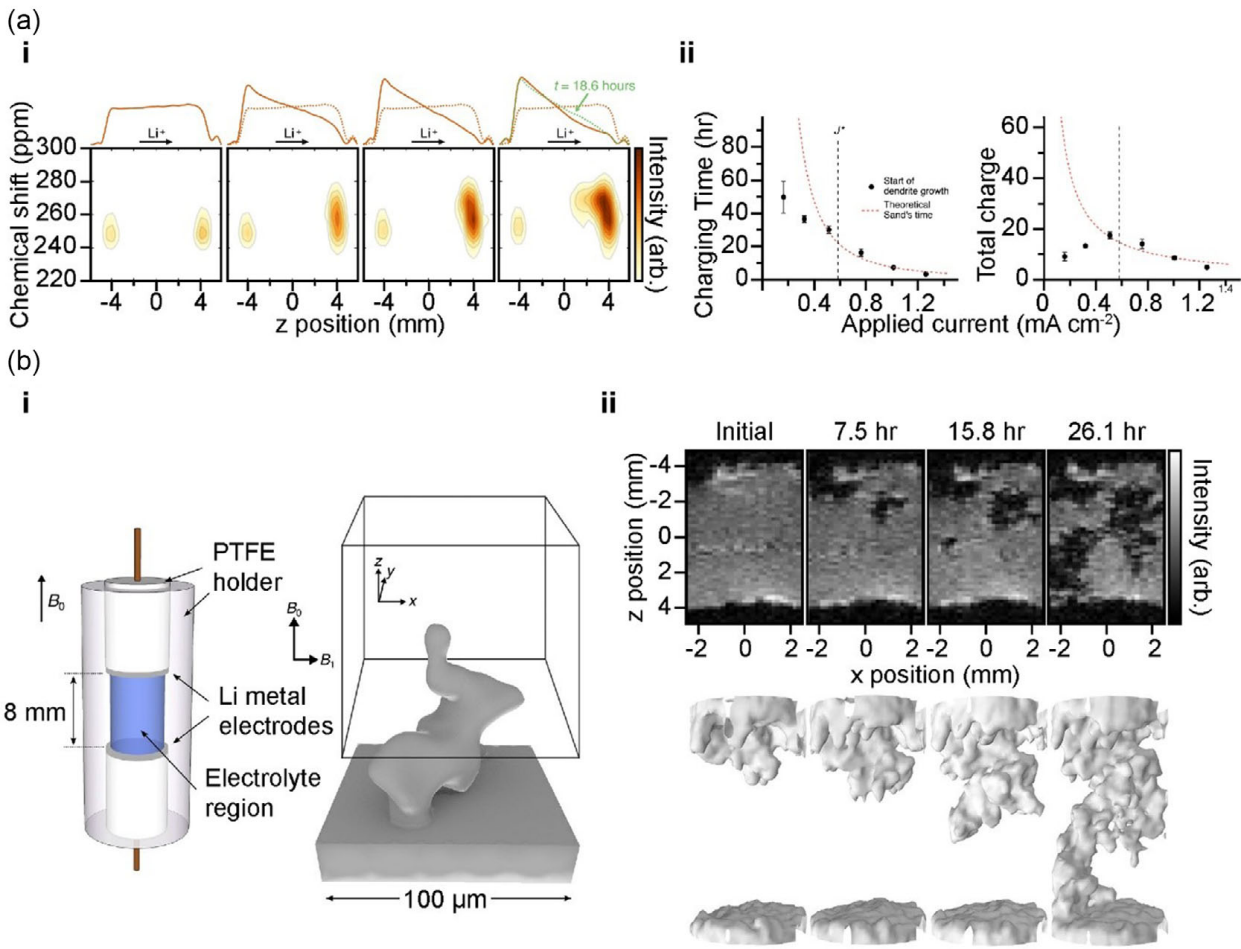
a) i) Evolution of ^7^Li concentration profile (top) and ^7^Li chemical shift images (bottom) for the cell charged at 0.76 mA cm^−2^. ii) Comparison of the theoretical Sand's time and the initiation time of dendrite growth obtained by magnetic resonance imaging (MRI) analysis. a) Reproduced with permission.^[^ ^74^ ^]^ Copyright 2015, American Chemical Society. b) i) Scheme of the electrochemical cell and the model dendrite used for the calculations, with the box drawn to illustrate a given MRI voxel position around the dendrite. ii) In situ ^1^H 3D FLASH imaging results (acquired with time interval, 16 min 40 s) with each 3D reconstruction. 2D slices (top) and 3D segmented images smoothen with a Gaussian filter (bottom). b) Reproduced with permission.^[^ ^75^ ^]^ Copyright 2016, The Authors, published by National Academy of Sciences, USA.

The ^7^Li NMR analysis provides valuable insights into the behavior of batteries. Furó et al. at the Royal Institute of Technology used ^7^Li NMR to study the Li‐ion concentration distribution in an electrolyte under inducing current, by reproducing the state of Li plating in a customized Li symmetric cell installed in an NMR tube. The concentration profile was obtained for Li ions in their spatial and temporal evolution in the electrolyte, visualizing the concentration gradient in the evolution under the impact of a current and identifying the increased unevenness in Li concentration distribution across electrodes with increased current density.^[^ ^73^ ^]^ Additionally, Grey et al. at Cambridge University used ^7^Li NMR to visualize the dendrite growth in a Li‐metal battery and the Li‐ion concentration gradient in an electrolyte, leading to the visualization of the micromorphology of Li plating in line with changes in the current density on the surface of Li (**Figure** **6**a). The correlation between the time of dendrite formation and that of Li‐ion depletion on the surface can be identified using this technique.^[^ ^74^ ^]^ Furthermore, Jerschow et al. utilized ^1^H MRI to visualize dendrite growth by measuring the interactions between the Li‐metal electrode and electrolyte using a customized cell for radio frequency (RF) transmission (Figure 6b). MRI allows for the rapid generation of 3D images and the quantification of dendrites based on their indirect effects on the surrounding electrolyte (rather than via the detection of the Li‐metal nucleus).^[^ ^75^ ^]^ Despite the practical limitations of NMR and MRI, such as the need for specialized equipment and expertise and the relatively long measurement time, it remains a valuable tool for understanding battery behavior, particularly in the context of electrolyte‐ and dendrite‐related phenomena.

Electrode‐level imaging techniques, such as X‐ray‐based imaging, neutron radiography and tomography, and MRI analysis, have proven to be powerful tools for studying battery failure mechanisms by enabling the visualization of chemical and structural changes within battery components, allowing the identification of failure modes and the development of strategies to mitigate them. However, these techniques have practical limitations in that they require specialized equipment and expertise. For instance, X‐ray‐based imaging techniques require expensive synchrotron radiation facilities or X‐ray sources, and the interpretation of X‐ray diffraction patterns and other imaging data requires expertise in crystallography and material characterization. Similarly, neutron radiography and tomography require access to a neutron source and expertise in neutron scattering and imaging techniques to interpret imaging data.

Additionally, electrode‐level imaging studies require expertise in experimental design and sample preparation, such as developing customized cells for battery analysis and considering the cell geometry, electrical connections, and compatibility with the imaging technique. Sample preparation also requires careful electrode and electrolyte handling to prevent contamination or damage to battery components.^[^ ^72^, ^76^, ^77^, ^78^, ^79^, ^80^, ^81^ ^]^ Another practical limitation of electrode‐level imaging is the potential of imaging techniques to cause damage to battery components, such as structural changes and damage to the electrode and electrolyte materials, leading to inaccurate results.^[^ ^62^, ^81^ ^]^ Therefore, careful consideration and optimization of imaging conditions are necessary to minimize the impact of imaging on battery performance. The global visualization of a cell's internal structure is another important aspect of battery analysis. The next chapter explores the various imaging techniques used for internal battery structure visualization, their applications, and the insights they provide into battery behavior and performance.

## 4 Visualization of the Battery Internal Structure

**Figure 7:**
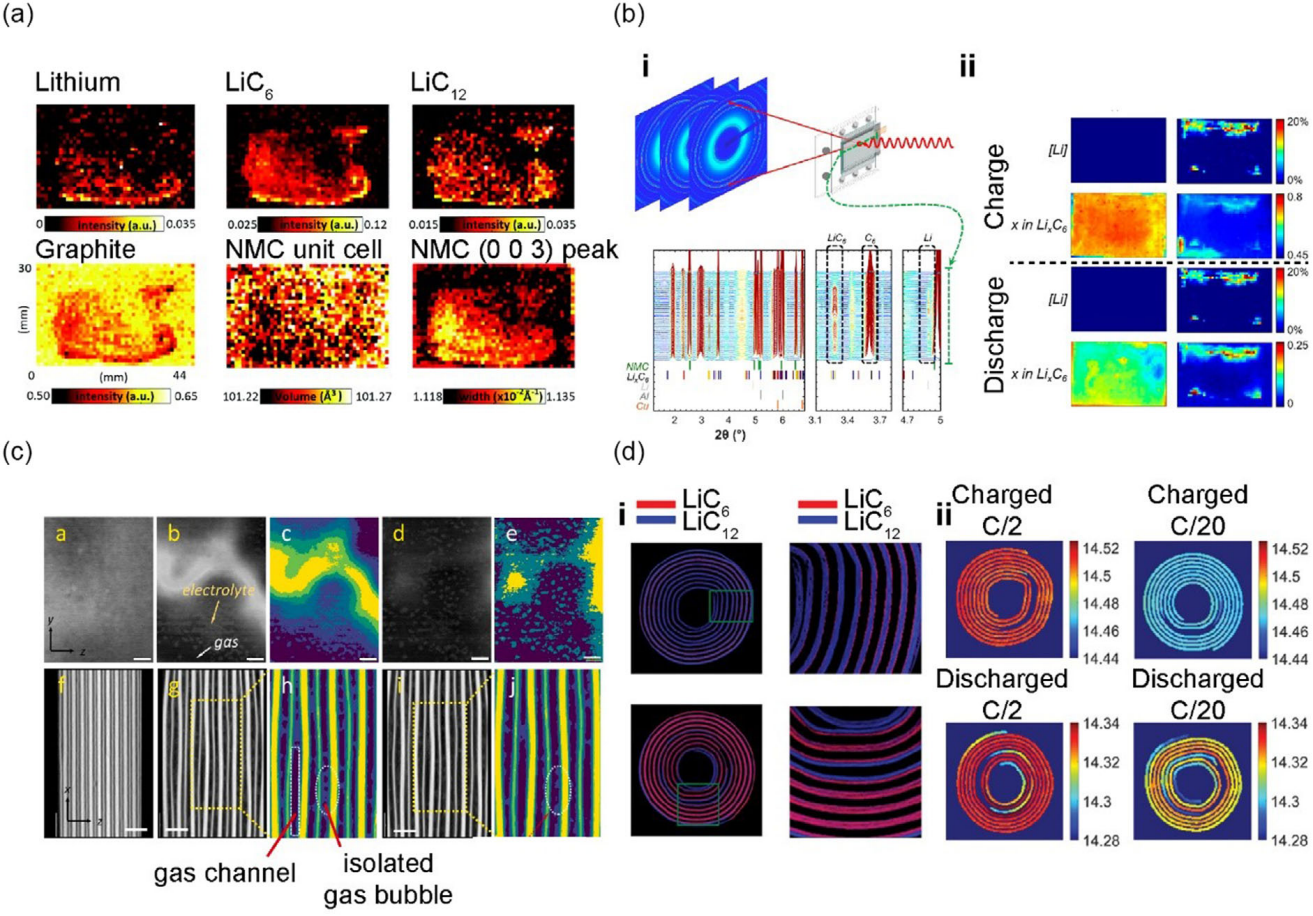
a) Spatial maps of irreversibly plated Li, the anode, and cathode phase, obtained through XRD. Reproduced with permission.^[^ ^82^ ^]^ Copyright 2021, Royal Society of Chemistry. b) i) Schematic of the experimental setup and set of XRD patterns collected along the green dashed line as indicated. ii) Color maps of heterogeneous distributions at 0 (range from 0.65 to 1 at charge) and 1255 cycles. b) Reproduced with permission.^[^ ^83^ ^]^ Copyright 2021, American Chemical Society. c) Time‐lapse X‐ray tomographic slices of Li‐ion pouch cell at each cycle: front (a–e) and side (f–j) views of slices (green: Cu, yellow: cathode with Al, blue: polymer electrolyte, and purple: gas). c) Reproduced with permission.^[^ ^84^ ^]^ Copyright 2022, Elsevier. d) Synchrotron high‐energy XRD CT: i) phase distribution maps of the crystalline phases in the anode and ii) lattice parameter *c* (Å) maps for the primary hexagonal NMC532 phase. d) Reproduced under the terms of the CC‐BY Creative Commons Attribution 4.0 International license (https://creativecommons.org/licenses/by/4.0).^[^ ^85^ ^]^ Copyright 2021, The Authors, published by Wiley‐VCH.

The overall battery states of large‐scale commercial cells should be macroscopically monitored because of the uneven internal reactions, pressure, and current distributions caused by defects related to the manufacturing process and degradation. Unlike the previously described imaging analyses concerning the active material and electrode units, the analysis of commercial batteries should exhibit a high level of X‐ray energy to allow for the penetration of packaging materials and rapid scanning of large areas. Therefore, studies on the visualization of battery structures have applied high‐speed scanning using high‐energy synchrotron X‐rays. Weker et al. used high‐energy XRD to visualize plated Li and the local SOC on an electrode during fast charging.^[^ ^82^ ^]^ Specifically, XRD was used to characterize the local lithiation after 450 cycles of fast charging of a 3.1 × 4.5 cm^2^ single‐layer pouch cell. Through *mm*‐scale imaging, the correlations between irreversible lithiation at the graphite anode, the inactive graphite phase, and local SOC were identified. Notably, the 2D map obtained using XRD allowed for the quantification of the correlations between SEI growth, Li plating, and loss via graphite‐trapped Li (**Figure** **7**a). A high‐energy synchrotron X‐ray was used by Ren et al. to detect and quantify Li plating in a single‐layer pouch cell.^[^ ^83^ ^]^ In this study, the total area of the cell was scanned using X‐rays and after image conversion, the Li and Li_*x*_C_6_ components were mapped (Figure 7b). Thus, the location of lithiation in the pouch cell after the onset of the fast‐charging cycle can be identified. During the extended cycle life, the concentration of metallic Li followed a sigmoidal curve that indicated a two‐stage continuous nucleation and autocatalytic growth, whereas the efficiency of Li stripping showed an exponential decay with increasing cycle life due to the accumulation of metallic Li.

Shearing et al. used high‐energy X‐ray CT to noninvasively visualize the structural evolution caused by gas formation after cycling a commercial Li‐ion pouch cell.^[^ ^84^ ^]^ They reported gas formation leading to battery transformation on a macroscopic scale at 4D (3D + time) and the mechanism of the internal gas evolution of the electrode upon cycling (Figure 7c). They examined the time‐lapse 3D thickness distribution at different locations in the pouch cell (front, middle, and back), highlighting the existence of an imbalance in gas formation. Vamvakeros et al. used high‐energy synchrotron X‐ray CT to monitor the spatial state of imbalance in a 600 mAh cylindrical battery during a charge–discharge process.^[^ ^85^ ^]^ Through this, the chemical species and crystallinity of a cross‐section of the battery were visualized at a high spatiotemporal resolution of 20 × 20 × 3 μm^3^ voxel for 1 h. Thus, the Li_*x*_C phase at the graphite anode and uneven distribution of the lattice constants of the nickel–manganese–cobalt (NMC) cathode material could be visualized for the area of the central jellyroll axis in the battery design during the charge–discharge process (Figure 7d).

The combination of multiple imaging techniques is powerful for comprehensive cell inspection. The Liu team at SLAC employed advanced tools, such as multiscale X‐ray tomography, SEM–Raman spectroscopy, and synchrotron‐based analytical techniques, to identify and picture diverse structural flaws in 18 650‐type cells.^[^ ^86^ ^]^ Upon performing a comprehensive synchrotron‐based analysis of the defective areas on an unrolled electrode removed after battery cycling, the team discovered a variety of structural defects. These include: 1) a misaligned Cu current collector near the positive terminal of the cell; 2) burrs on the anode tab; 3) sporadically and thinly spread material impurities; 4) uneven active particle packing; and 5) electrode delamination. Notably, they reported that the localized electrochemical redox disparity, driven by preexisting metallic impurities, could potentially intensify, leading to an accelerated degradation of the electrode.

**Figure 8:**
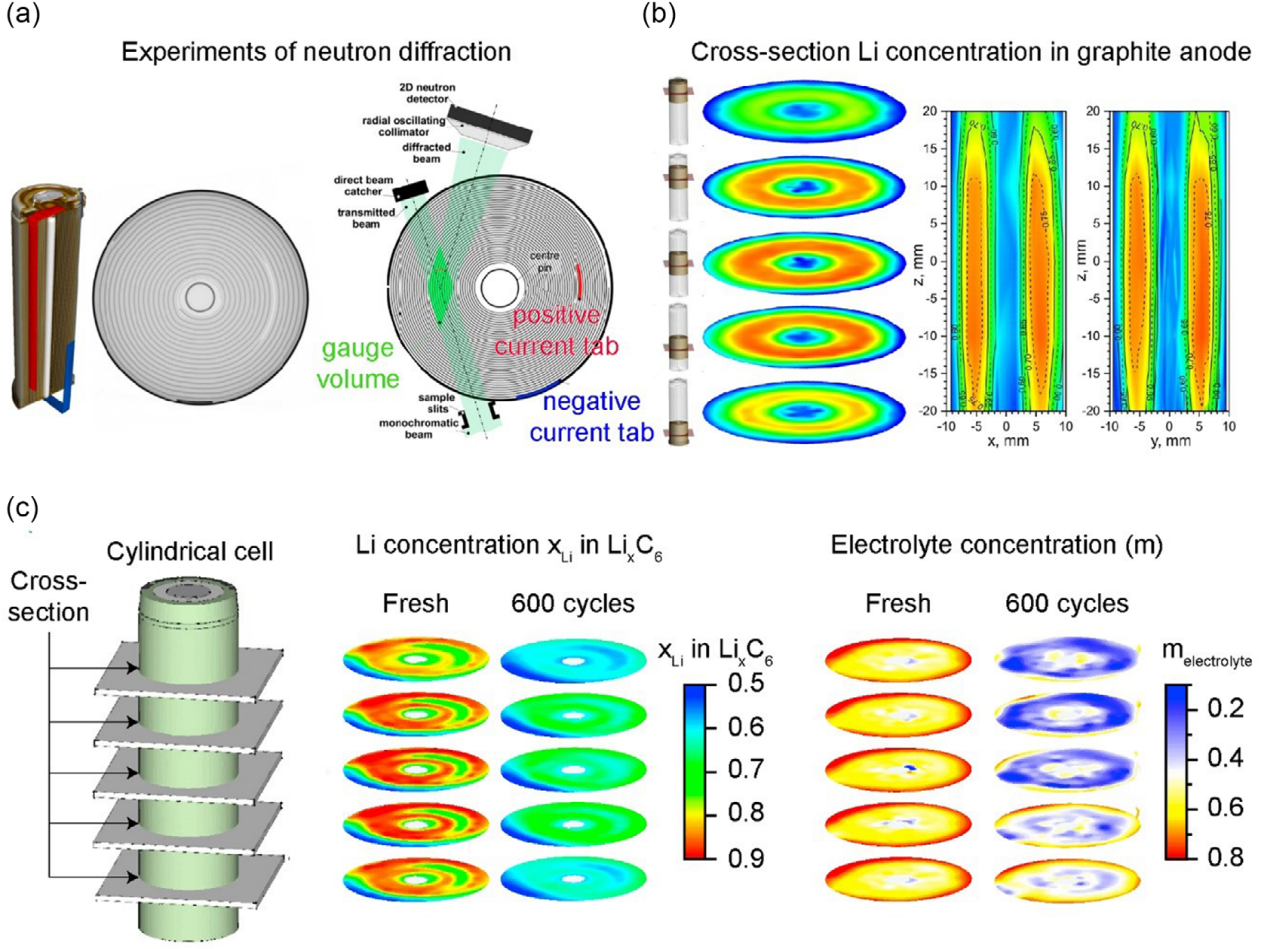
a) Spatially resolved neutron diffraction experiments on 18 650‐type Li‐ion cell. Typical layout of the electrode and current tabs and scheme of the gauge volume. Reproduced with permission.^[^ ^88^ ^]^ Copyright 2020, Elsevier. b) Neutron imaging of Li concentration in graphite anode. Li concentration in Li_*x*_C_6_ at different heights and reconstructed vertical Li distribution along the cell. Reproduced under the terms of the CC‐BY Creative Commons Attribution 4.0 International license (https://creativecommons.org/licenses/by/4.0).^[^ ^87^ ^]^ Copyright 2015, The Authors, published by Springer Nature. c) Li and electrolyte concentrations at different planes of fresh and aged (600 cycles) of 18 650 cylindrical cells. Reproduced with permission.^[^ ^88^ ^]^ Copyright 2020, Elsevier.

Neutron diffraction and/or radiography are also effective for the macroscopic evaluation of Li concentrations and the evenness of electrolyte distributions in commercial batteries (**Figure** **8**a). Senyshyn et al. visualized the Li distribution at a graphite anode according to the tab design of a cylindrical LIB based on neutron scattering (Figure 8b).^[^ ^87^ ^]^ A high level of lithiation was detected at the anode close to the tab, owing to the linear increase in resistance up to the current collector at a location distant from the tab. This finding highlights the importance of designing tabs to ensure evenness of the current flowing through the current collector. They further used neutron diffraction to visualize the amount of Li in a graphite electrode and the electrolyte distribution in a commercial battery (Figure 8c).^[^ ^88^ ^]^ Before cycling, the electrolyte was found to be mainly collected at the outer part of the cell, leading to a higher level of lithiation at the graphite anode in the outer area of the battery after cycling. In addition, electrolyte accumulation at the bottom of the cylindrical battery was found to cause uneven Li distribution in the direction of the battery height. Siegel et al. used neutron radiography to visualize variations in electrode thickness according to changes in the Li concentration and charging state in a pouch cell and showed an irreversible increase in volume and capacity fade related to uneven Li insertion in the anode at a high rate of charging.^[^ ^89^ ^]^

Neutron‐based radiography provides a unique way to visualize materials that is distinct from X‐rays and often serves as a complementary method. X‐rays primarily interact with the electron shell, whereas neutrons interact with nuclei. This fundamental distinction in the physical principles of interaction means that neutrons are significantly attenuated by certain light elements such as H, Li, B, whereas metallic elements such as Al have minimal impact on the beam intensity.^[^ ^90^ ^]^ Hence, the mass attenuation coefficient of hydrogen groups in liquid electrolytes distinguishes them from other cell materials and aluminum. Hydrogen groups have a higher coefficient, making the liquid appear darker, whereas dry spots appear brighter in the neutron radiography images.

Using this feature, Knoche et al. at the Technical University of Munich in situ visualized the spatial distribution of liquid electrolytes within pouch cells and first examined how sealing the cell and venting the vacuum chamber affect the electrolyte filling process^[^ ^77^ ^]^ by exploiting the ANTARES facility at the Heinz Maier–Leibnitz Zentrum in Garching, Germany. They found that the electrolyte permeated in a bottom‐to‐top manner, necessitating unobstructed pathways for the escape of trapped gases. They also stated that excess electrolyte above the cell stack should be avoided to prevent the entrapment of gas bubbles and ensure a well‐regulated electrolyte flow within the electrode assembly through precise injection, optimal needle positioning, and thoughtfully engineered cell design. The factors determining the electrolyte wetting quality were further investigated by varying the pressure and number of deputy wetting cycles. Weydanz and Furth et al. in situ visualized electrolyte filling for hard‐case prismatic cells with Al housings under various pressure conditions.^[^ ^91^ ^]^ The key finding was that the electrolyte solution simultaneously penetrated the electrode stack from all four sides at almost the same speed as the electrodes toward the center. The height of the cell determines the total wetting time of the stack and should be small, whereas the number of electrodes has no impact on the wetting process. This study provides valuable insights into optimizing the production process and designing an optimal cell geometry.

Although X‐ray tomography, neutron diffraction, and radiography are successful techniques for scanning batteries, their application to actual fields in the battery industry is limited, as processing capacity for a greater amount of data and a relatively long period are required to investigate the overall area of a battery. Acoustic wave imaging has recently been introduced and has received considerable attention as a promising method for the noninvasive characterization of battery operation throughout its longevity. Acoustic wave imaging is an effective technique for analyzing a battery's internal components and is based on the characteristic rapid attenuation of the ultrasonic transmission rate and amplitude according to the properties of the medium (coefficient, density, thickness, and porosity). For example, Steingart et al. at Princeton University scanned various cells (pouch, cylindrical 18 650, and alkaline AA cells) to measure the time of flight (the time required for an ultrasound wave to traverse the medium).^[^ ^92^ ^]^ The cycle‐dependent time‐of‐flight profile and variations in the ultrasound amplitude were key indicators for detecting changes in the stress, transformations of the cathode material, and SEI formation on the anode active material. These correlations imply that acoustic wave imaging can be used to assess a battery's state of health (SOH).

**Figure 9:**
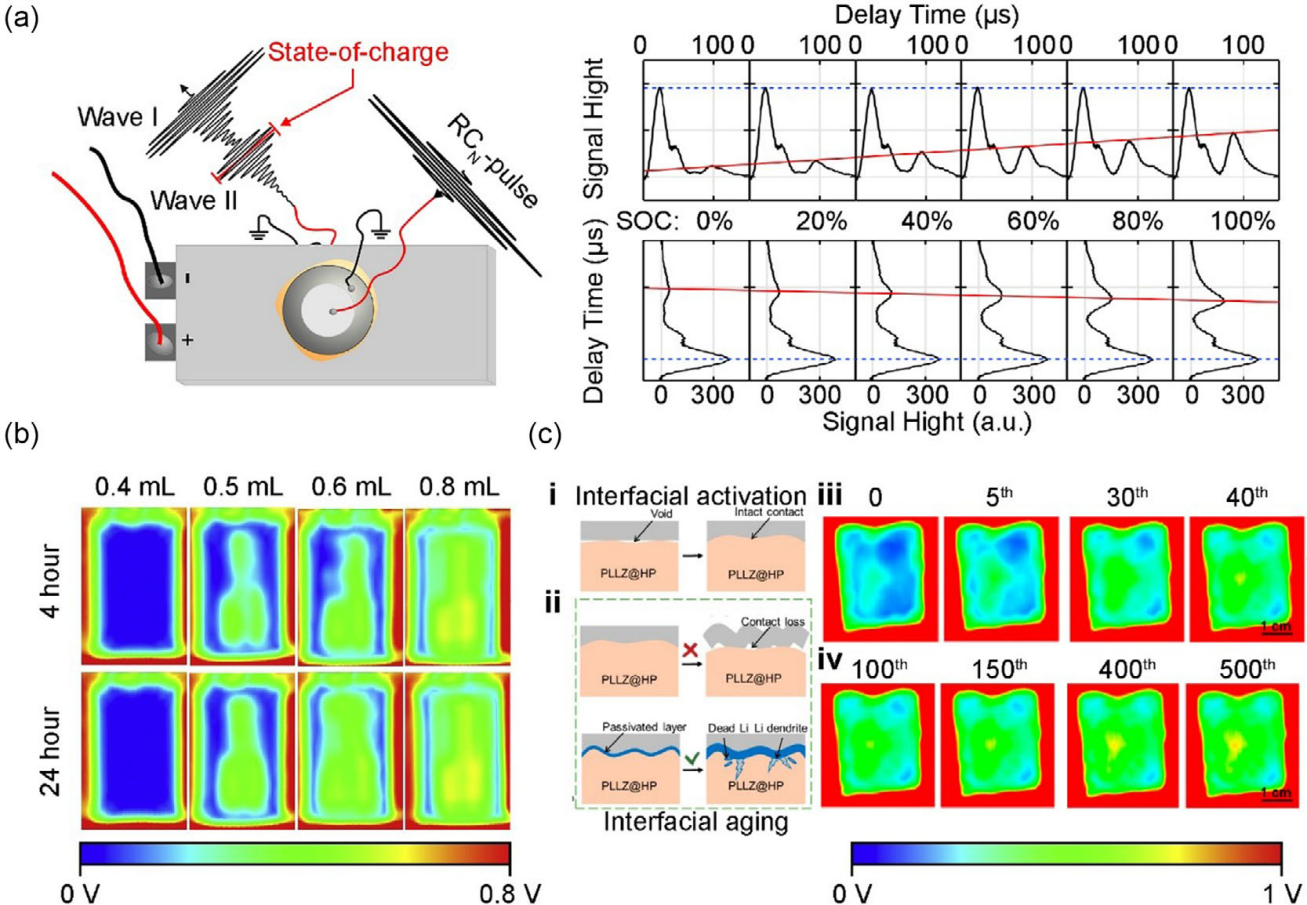
a) Smoothed modulus of the transmitted signal of RC_*N*_‐pulses through a Li‐ion pouch cell. Stepwise (20%) SOC change at 2 C charging. Linear dependence of slow wave's (solid red line) delay time on SOC. Reproduced with permission.^[^ ^93^ ^]^ Copyright 2017, Elsevier. b) Ultrasonic images of Li‐ion pouch cells with different volumes of electrolyte after wetting for 4 and 24 h. Reproduced with permission.^[^ ^95^ ^]^ Copyright 2021, Elsevier. c) Scheme of i) the interfacial activation process, ii) a possible mechanism for the cell overpotential increase, iii) corresponding ultrasonic transmission images of the Li/PLLZ@HP/Li cell during the first 40 cycles, and iv) during 100–500 cycles. c) Reproduced with permission.^[^ ^96^ ^]^ Copyright 2022, American Chemical Society.

Sextl et al. investigated the characteristics of ultrasonic transmission in battery pouch cells based on an ultrasound pulse.^[^ ^93^ ^]^ Whereas previous studies using acoustic wave imaging focused solely on measuring battery thickness and stress, this study considered the characteristic sensitivity of the variations in porosity at the graphite anode to the ultrasound pulse during the charge–discharge process. As a result, a linear correlation was found between the time of flight of the transmitted wave frequency and the battery SOC (**Figure** **9**a). Subsequent studies have investigated other methods for detecting internal battery defects using acoustic wave imaging. One example is the Steingart group, who reported the detection of Li‐metal plating after ultrasonic transmission scanning of the entire area of a 400 mAh LiCoO_2_/graphite pouch cell (3 × 2 cm^2^) during charging.^[^ ^94^ ^]^ Attenuation of the ultrasound amplitude was observed in areas close to the tab during fast charging, which was attributed to Li‐metal plating due to the high current density near the tab. Moreover, through differential amplitude analysis, the level of graphite lithiation was visualized by combining the acoustic signals and the graphite phase transformation.

Dahn et al. used ultrasonic scanning of a Li‐ion pouch cell to visualize the distribution of the internal electrolyte impregnation of the battery.^[^ ^95^ ^]^ A thin beam (diameter of 1 mm) was used to obtain high‐resolution ultrasonic scanning images below the submillimeter (sub‐mm) scale. Electrolyte impregnation has been reported based on the characteristic attenuation of the ultrasound amplitude according to the media, such as the internal solid of the pouch cell (active material), liquid (electrolyte), vacuum, or gas (electrolyte nonimpregnation and gas formed via the reaction between the electrolyte and active material) (Figure 9b). In addition, large‐scale pouch cells (130 × 310 mm) and rectangular cells (120 × 190 mm) were analyzed to verify their potential use in commercial cells. However, there was a limitation, in that the measurement could only be performed in liquids (silicon oil) to induce amplitude reduction in the gas‐phase ultrasound.

Huang et al. successfully visualized gas emissions in a solid‐state battery pouch cell (20 × 30 mm) and interface degradation during cycling (Figure 9c). This was accomplished using an operando method with a high sensitivity of the ultrasound signal to gas or vacuum.^[^ ^96^ ^]^ For the first 40 cycles, the blue areas in the image turned green, indicating an improvement in the interfacial contact between the Li metal and the solid electrolyte. In addition, based on the electrochemical results of a gradual increase in the overpotential during long‐term cycling, two mechanisms of degradation were suggested: loss of contact due to 1) the change in the volume of Li metal and 2) growth of the porous passivation layer. They experimentally identified the true mechanism using ultrasonic imaging, which confirmed a well‐contacted interface without void formation. Acoustic imaging offers the advantage of monitoring spatial variations based on the characteristic attenuation in amplitude depending on the presence or absence and type of medium. However, a limitation of acoustic imaging is its inability to differentiate between elements in materials influenced by such variations. Despite this limitation, the high sensitivity to gas or vacuum and relatively fast scanning time of acoustic imaging are highly feasible to assess internal battery gas formation and distribution, evenness of electrolyte impregnation, and changes in internal porous structures that do not require high‐resolution elemental analysis.

## 5 Visualization of Battery Current Distribution

### 5.1 Thermal Imaging of Li‐Ion Pouch Cells

**Figure 10:**
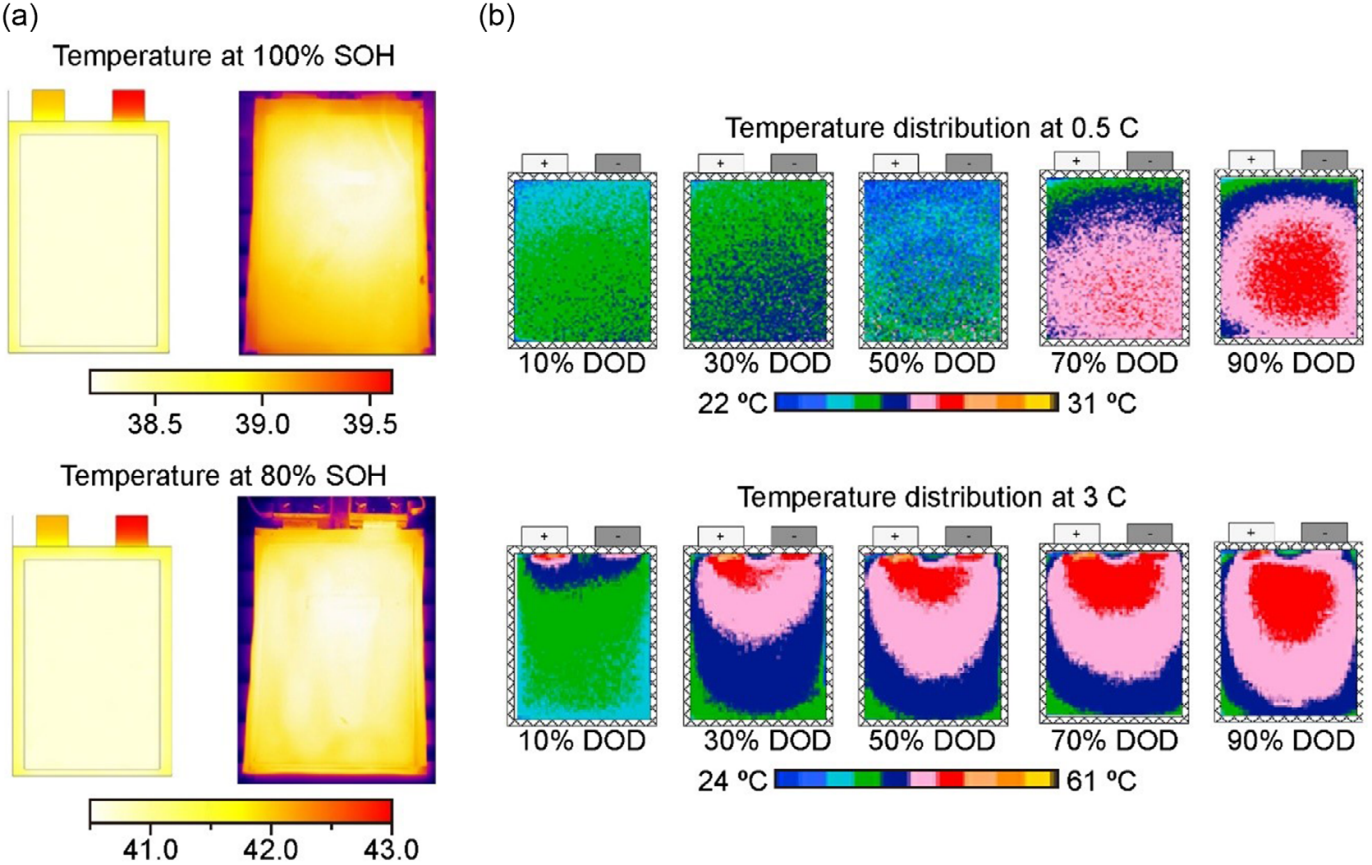
a) Cross‐validation of temperature distributions with 3D modeling results (left) and infrared radiation (IR) thermal pictures taken by Ti25 FLIR thermal camera (right) at 100% and 80% SOH of 20 Ah‐class pouch cells cycled under the fast‐charging conditions. Reproduced under the terms of the CC‐BY Creative Commons Attribution 4.0 International license (https://creativecommons.org/licenses/by/4.0).^[^ ^97^ ^]^ Copyright 2018, The Authors, published by MDPI. b) Color development guided by infrared imaging for visualizing the temperature distribution of 37 Ah‐class pouch cells (*T* 7.1 mm × *H* 268 mm × *W* 211 mm) at a discharge rate of 0.5 C and 3 C at depth‐of‐discharge (DOD) of 10%, 30%, 50%, 70%, and 90%, respectively. Reproduced with permission.^[^ ^98^ ^]^ Copyright 2019, Elsevier.

Efforts have been taken in applying thermal infrared imaging to measure the heat generated according to the flow of a battery's internal current from the reversible heat (the entropic component) and irreversible heat (the component representing the ohmic and polarization resistance). By cross‐validation of infrared camera‐guided thermal imaging and 3D modeling, De Hoog et al. visualized the SOH‐dependent temperature distributions of commercial pouch cells cycled under fast pulse charging condition (20 Ah).^[^ ^97^ ^]^ Of note, when the cell aging was accelerated by fast‐changing cycling, clear difference of infrared‐driven thermal imaging can be visualized, indicating the nonuniform distribution at the aged cell (SOH 80%). They also stated the interactions between the thermal and electrical properties of cells during prolonged cycling (**Figure** **10**a). The actual measured temperature of cells with degradation showed an accuracy level within a range of ±2 °C error relative to the simulation results, verifying the potential use of the suggested modeling for a battery management system (BMS). The heat generation and distribution of the cells can be affected by applied current densities and the depth of discharge (DOD). Wang et al. visualized thermal behaviors of NMC‐type Li‐ion pouch cells at various discharge rates and DODs (Figure 10b).^[^ ^98^ ^]^ Therefore, it was possible to identify the local area of the heat accumulation and concentration increases rapidly and roughly estimate the degree of cell degradation.

Even though infrared imaging is simple and effective to investigate the cell imbalances in heat dissipation, nonuniform temperature distribution observed in the above studies is possibly attributed to different thermal conductivities across the active materials and current collectors, not triggered by internal defects and/or states of the cells. Moreover, Swierczynski et al. empirically verified that the surface temperature distribution of the cell would not represent the actual cell temperature and less relevant with the SOC.^[^ ^99^ ^]^ Even though the location where heat excessively generated by the exothermic side reactions could be roughly identified, it remains challenging to pinpoint or trace such defect spots using infrared imaging studies. Therefore, infrared‐guided thermal imaging should be further validated for determining the state of battery and providing the information of internal defects. Instead, it is suggested to perform the in situ infrared imaging upon thermal runaway of the batteries, which seems to be rather effective to identify the “hot spots” that initiate the self‐heating before the thermal runaway.

One more consideration is that high throughput of imaging analysis is the key to fast cell inspection. For instance, the X‐ray irradiation time for a single scan can be very short. Nonetheless, it is not preferred to quickly seek the defect spots over the whole cell owing to lower contrast greyscale images, and more time (a few hours) might be needed to reconstruct a larger number of images and render a 3D tomogram. Even though high‐speed (≈Hz) synchrotron radiation X‐ray tomography and radiography have been proposed,^[^ ^100^ ^]^ it still requires a large‐scale synchrotron beamline facility, hindering industrial applications. In contrast, ultrasonic and infrared imaging can be used in‐factory and only take a few minutes for global imaging of commercial cells without additional image processing, which is more suitable for fast inspection and identification of defect spots. Indeed, the ultrasonic scan takes about 5 min for one scan for the 20 × 30 mm cell.^[^ ^95^ ^]^ Nonetheless, the application in the production line is still limited by the cumbersome that the cell must be placed in an oil bath. While heat imaging with an infrared camera or sensor features high throughput, it takes a time delay in warming up the battery to sense the Joule heat.

### 5.2 Magnetic Resonance Imaging (MRI) of Li‐Ion Pouch Cells

The MRI techniques described in Section 3.3 can be used for monitoring electrochemical properties during battery operation, such as electrolyte distribution and dendrite formation of Li metal. However, the limitation regarding the nontransmission of RF signals in various conductive components of batteries has generally prevented the use of these techniques in the study of commercial batteries. Hence, all the previously described studies were conducted by producing customized cells to allow for RF irradiation. This led to the development of a technique for diagnosing batteries without internal RF irradiation.

**Figure 11:**
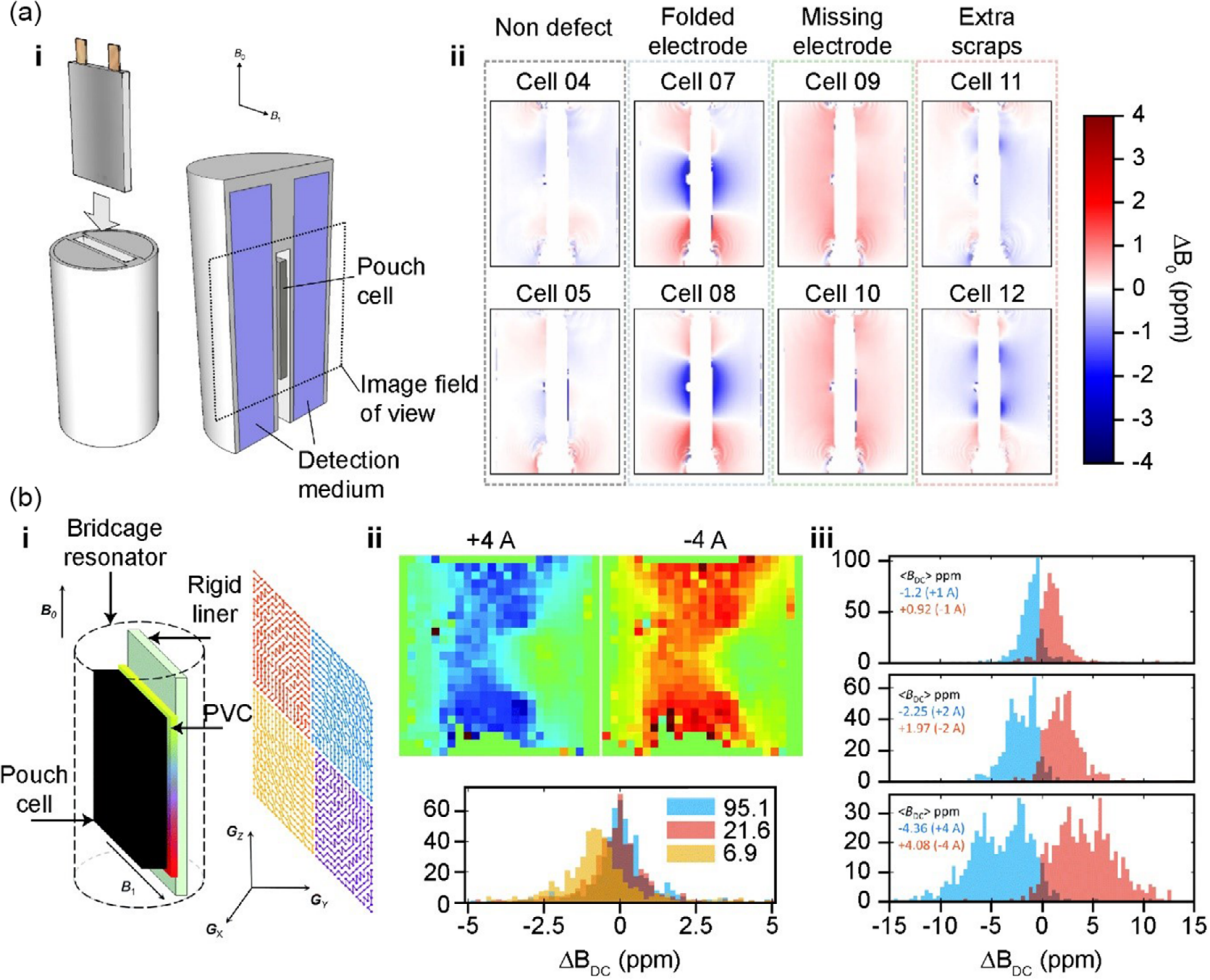
a) i) Magnetic resonance imaging (MRI) measurement setup for the Li‐ion pouch cells. ii) MRI maps for the defective cells relative to defect‐free control cells with the mean and standard deviation. a) Reproduced under the terms of the CC‐BY Creative Commons Attribution 4.0 International license (https://creativecommons.org/licenses/by/4.0).^[^ ^101^ ^]^ Copyright 2018, The Authors, published by Springer Nature. b) i) Scheme of surface‐scan MRI with 2D centric‐scan single‐point ramped imaging with T1 enhancement (SPRITE) sampling. ii) MRI maps of magnetic field distribution produced by a fully charged cell (Nokia BL‐5C). iii) Histograms of field breakdown coefficients (BDCs) distribution demonstrating effects of the operation mode and the current magnitude. Average field BDC values are indicated on each plot. b) Reproduced with permission.^[^ ^104^ ^]^ Copyright 2021, Royal Society of Chemistry.

Jerschow et al. of New York University (who applied ^1^H MRI in the analysis of electrodes) used MRI to visualize the changes in magnetic susceptibility according to the Li concentration in the cathode material, and consequently induced changes in the magnetic field surrounding the battery. The results were used to SOC determination and diagnosis of internal defects in the battery assembly process (**Figure** **11**a). The conventional MRI approach necessitates the production of a customized cell (as RF transmission is disallowed through conductors); however, this new technique allows for MRI measurements of the interior of commercial cells without RF transmission. The cell was placed in a holder filled with water, and the influence of the battery on the water molecules was measured. Thus, the changes in the magnetic susceptibility according to the different SOCs coincided with the pattern of change in the Li concentration in the cathode. However, this technique is limited in identifying the types and locations of battery defects and in placing a commercial cell in a customized holder filled with water.^[^ ^101^ ^]^

Furthermore, MRI in magnetic field measurements allows the visualization of the current distribution by detecting the magnetic field induced by the current applied to the battery. In contrast to previous studies, in which the change in magnetic susceptibility was measured in accordance with the lithiation state of the electrode active material, the magnetic field induced by the applied current was visualized using MRI. The current distribution between the charge and discharge cycles in a defective battery displayed asymmetry, and the SOC determined the distribution of the magnetic field surrounding the battery (Figure 11b).^[^ ^102^ ^]^ In addition, a follow‐up study introduced the method of single‐point ramped imaging with T1 enhancement (“SPRITE”), aiming to solve the problems concerning low sensitivity and noise in conventional MRI for batteries. This allowed for rapid and accurate visualization of the magnetic field distribution. As a result, the magnetic field induced in a 1.8 Ah commercial battery was visualized, and magnetic field distribution pattern variations and asymmetry were observed for a defective battery with an external short circuit.^[^ ^103^ ^]^ However, low sensitivity persists, posing limitations for accurate measurements of the magnetic field pattern. To improve this, a novel method (surface MRI scan) was introduced to allow the pattern to be accurately assessed. In particular, the measurements of the magnetic field were taken on a thin solid layer in contact with the battery (Figure 11c). Using this technique, an MRI can detect a local imbalance in the current density and its location in the battery.^[^ ^104^ ^]^

### 5.3 Magnetic Field Imaging (MFI) of Li‐ion Pouch Cells

**Figure 12:**
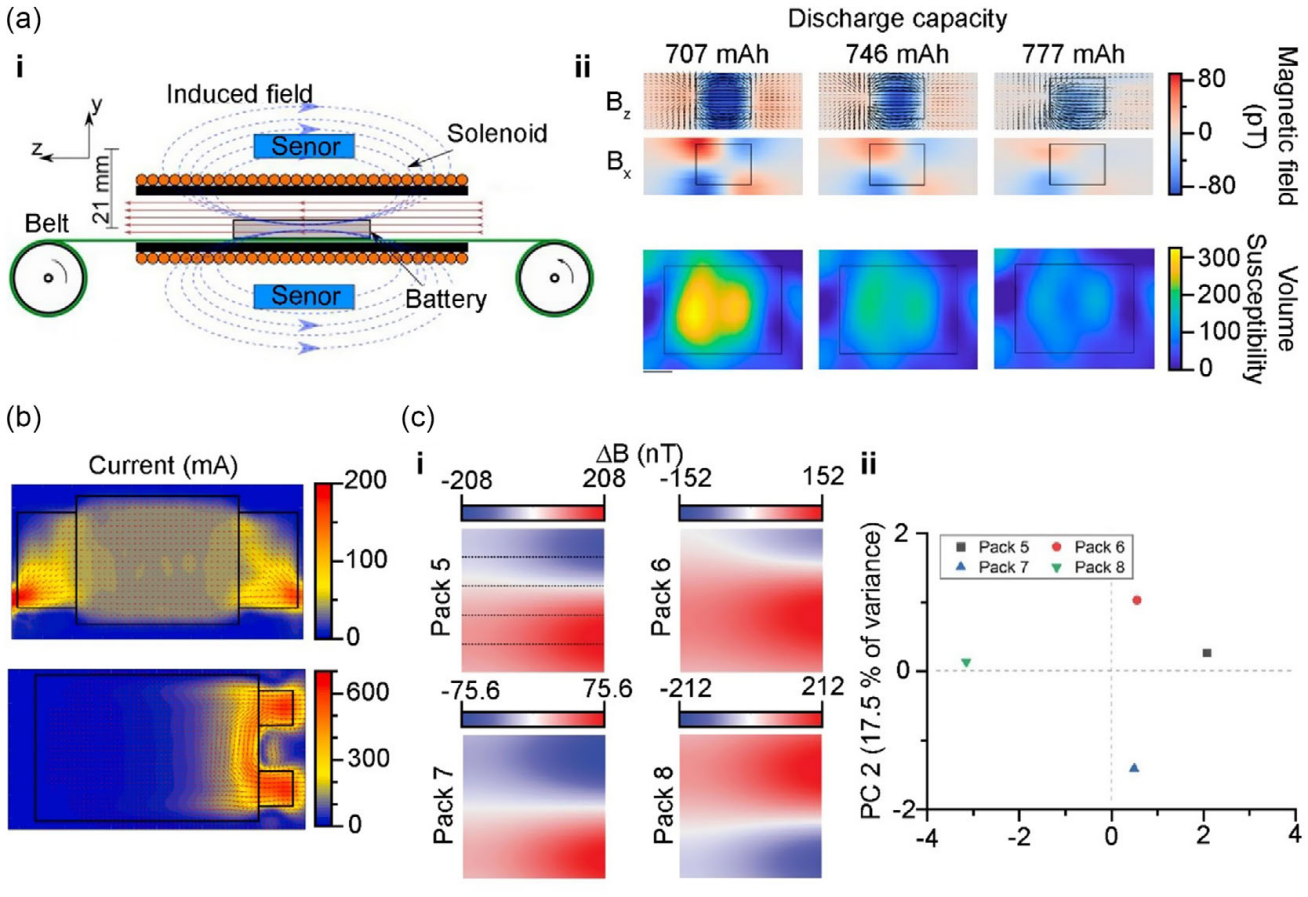
a) i) Scheme of the experimental arrangement for measuring magnetic field. Magnetic field sensors are placed in the ultralow‐field region, above, and below the solenoid. ii) Magnetic field and corresponding susceptibility maps above a cell. 2D magnetic susceptibility maps obtained from a regularized inversion of the measured magnetic field. a) Reproduced with permission.^[^ ^106^ ^]^ Copyright 2020, The Authors, published by National Academy of Sciences, USA. b) Reconstituted current distribution from the measured magnetic field of same‐side tab design and counter‐side tab design cells. Reproduced with permission.^[^ ^49^ ^]^ Copyright 2021, Elsevier. c) Magnetic field variation caused by the different current change between multicells. i) Magnetic field maps of the four cells with different current variations. The dashed lines indicate the positions of the cells. ii) Principal component analysis (PCA) results for the maps. c) Reproduced with permission.^[^ ^108^ ^]^ Copyright 2022, Wiley‐VCH.

The previously described MRI techniques for visualizing the internal battery current distribution by measuring the magnetic field distribution are economically disadvantageous owing to the high cost of the device. In addition, there is still a limitation regarding the device complexity in analyzing commercial cells, as the current must be applied to a customized holder. Infrared spectroscopy based on Joule heat is relatively simple and offers data on the distribution of current intensity;^[^ ^105^ ^]^ however, the spatial resolution is low, and there is a limitation in collecting data regarding current directionality. To overcome these problems, studies have investigated faster and simpler methods for visualizing a battery's internal current distribution using magnetic fields induced by the internal battery current. Jerschow et al. used an atomic magnetometer to measure and visualize the magnetic field surrounding a battery (**Figure** **12**a). Notably, as in the studies applying MRI to magnetic fields, the distribution of magnetic susceptibility over the whole area of commercial cell was visualized. Thus, an imbalance in the magnetic susceptibility due to variations in the local charging state was detected, and uneven Li intercalation/deintercalation on the electrode surface was experimentally verified.^[^ ^106^ ^]^

Kimura et al. used a magnetic sensor to visualize the distribution of magnetic fields induced by the flowing current in an LIB charge–discharge process. Notably, by measuring the LIB‐induced magnetic fields, it was also possible to visualize the ion diffusion rate of the electrolyte and separator and the reaction rate distribution for the active material. Accordingly, the expansion of the areas of abnormal conductivity was confirmed based on the changes in the uneven internal battery conductivity during cycling.^[^ ^107^ ^]^ Brauchle et al. of Daimler AG used an anisotropic magnetoresistive (AMR) sensor to measure the magnetic field surrounding a battery, as induced in the battery charge–discharge process, to visualize the current flow inside the batteries (Figure 12b). Notably, an AMR sensor generally has linear sensitivity across a wide range of magnetic fields, with the advantage of allowing the measurement and visualization of the magnetic field and current distribution, while avoiding the magnetic field shielding effect. Thus, the current distribution can be visualized by measuring the battery‐induced magnetic field, and the batteries with bidirectional or unidirectional tabs coincide uniformly with the simulation results.^[^ ^49^ ^]^

Magnetic sensor analysis of the magnetic field and current distribution can be applied to detect defects in a pack containing several batteries, beyond the simple detection of imbalances and defects in a single battery. For instance, Wu et al. visualized the magnetic fields of a battery pack and detected an imbalance in the capacity across the batteries in the pack (Figure 12c). Through magnetic field scanning using magnetic sensors, the uneven current and magnetic susceptibility of the materials were identified owing to the imbalance in capacity across the batteries connected in parallel in the pack.^[^ ^108^ ^]^ As demonstrated, although MFI‐based analysis allows for the detection and quantification of the macroscopic current distribution and flow pattern, it accounts only for the current distribution dominantly at the conducting materials (i.e., the current collector) and perceives the target cell as a 2D object. This poses substantial limitations on data extraction concerning charge transfer in the direction of the battery depth.^[^ ^49^ ^]^ Therefore, it is forecasted that improvements in 3D imaging analysis and the incorporation of charge transfer via ions in the electrolyte will lead to a highly useful analysis tool.

## 6 Conclusion and Perspectives

Analogous to medical radiology, this review provides an overview of the techniques for visualizing batteries at different scales, from individual active material particles to complete battery packs. By utilizing visualization techniques appropriate for each scale, microscopic and macroscopic defects that can lead to battery failure can be detected. Furthermore, this review discusses the methods used for real‐time monitoring, interpretation, and identification of the mechanisms underlying battery degradation. X‐ray‐ and neutron‐based visualization techniques are highly effective in analyzing the microscopic structural and chemical evolution behaviors of active materials and their interfaces during battery operation, with qualitative and quantitative analyses offering high utility. However, the application of these techniques requires specific experimental designs and customized cells, which raises doubts regarding their use in commercial cells. Nonetheless, if these techniques are applied to commercial cells, they are expected to contribute significantly to increasing our understanding of the mechanisms of internal battery degradation.

For large‐scale commercial cells, it is dangerous to replicate degradation or explosions in experiments, making it necessary to identify latent defects and detect degradation behaviors in advance by analyzing wide areas in a minimum time under predicted operating conditions that could lead to degradation. X‐ray tomography and imaging are effective for the real‐time monitoring of the internal structural evolution of a cell under an applied current, with excellent resolution for specific elements and materials. However, interpretation may be limited to visible changes, and data on liquid or air states may be limited. Ultrasonic transmission imaging is intuitive for monitoring internal gas formation and distribution in a battery, electrolyte impregnation, and changes in porous structures, which do not require advanced elemental analysis. However, a limitation lies in its application to qualitative analyses is limited, as it is difficult to differentiate the elements responsible for the changes in materials. Therefore, it is necessary to understand the material characteristics and scale of the analysis target and combine imaging techniques for multifaceted interpretations.

Imaging the invisible internal current flow and distribution is expected to be useful for detecting latent defects and identifying causal factors. MRI, which utilizes magnetic fields to visualize the internal battery current distribution, is likely to prove useful for detecting patterns of change in the current distribution, such as locating defects and identifying their causes, from the perspective of battery design. Furthermore, once various battery failure scenarios are established, a differential analysis of the cause(s) of the imbalance can be performed, allowing for a more comprehensive understanding of the battery behavior and failure mechanisms.

To effectively apply MFI techniques for the detection of defects and performance diagnosis in actual commercial cells, it is necessary to reproduce specific defects caused by battery materials, components, design, or production in a commercial cell based on an understanding of battery degradation mechanisms. This should be followed by the selective collection and digitization of the resulting imaging data. By using a noninvasive technique with rapid inspection for macroscopic monitoring of the current distribution and establishing relationships among behaviors regarding chemical and structural degradation, it will be possible to detect latent defects under actual defect‐inducing conditions. Furthermore, it will be possible to identify their locations and provide a causal analysis for an early diagnosis.

## Conflict of Interest

The authors declare no conflict of interest.
